# Angelicin—A Furocoumarin Compound With Vast Biological Potential

**DOI:** 10.3389/fphar.2020.00366

**Published:** 2020-04-16

**Authors:** Camille Keisha Mahendra, Loh Teng Hern Tan, Wai Leng Lee, Wei Hsum Yap, Priyia Pusparajah, Liang Ee Low, Siah Ying Tang, Kok Gan Chan, Learn Han Lee, Bey Hing Goh

**Affiliations:** ^1^Biofunctional Molecule Exploratory Research Group, School of Pharmacy, Monash University Malaysia, Subang Jaya, Malaysia; ^2^Novel Bacteria and Drug Discovery Research Group, Microbiome and Bioresource Research Strength Jeffrey Cheah School of Medicine and Health Sciences, Monash University, Subang Jaya, Malaysia; ^3^Institute of Biomedical and Pharmaceutical Sciences, Guangdong University of Technology, Guangzhou, China; ^4^School of Science, Monash University Malaysia, Subang Jaya, Malaysia; ^5^School of Biosciences, Taylor's University, Subang Jaya, Malaysia; ^6^Medical Health and Translational Research Group, Jeffrey Cheah School of Medicine and Health Sciences, Monash University Malaysia, Subang Jaya, Malaysia; ^7^Institute of Pharmaceutics, College of Pharmaceutical Sciences, Zhejiang University, Hangzhou, China; ^8^Key Laboratory of Biomedical Engineering of the Ministry of Education, College of Biomedical Engineering & Instrument Science, Zhejiang University, Hangzhou, China; ^9^Chemical Engineering Discipline, School of Engineering, Monash University Malaysia, Subang Jaya, Malaysia; ^10^Advanced Engineering Platform, Monash University Malaysia, Subang Jaya, Malaysia; ^11^International Genome Centre, Jiangsu University, Zhenjiang, China; ^12^Division of Genetics and Molecular Biology, Faculty of Science, Institute of Biological Sciences, University of Malaya, Kuala Lumpur, Malaysia; ^13^College of Pharmaceutical Sciences, Zhejiang University, Hangzhou, China; ^14^Health and Well-Being Cluster, Global Asia in the 21st Century (GA21) Platform, Monash University Malaysia, Subang Jaya, Malaysia

**Keywords:** angelicin, psolaren, furocoumarin, biological activities, biological potential

## Abstract

Angelicin, a member of the furocoumarin group, is related to psoralen which is well known for its effectiveness in phototherapy. The furocoumarins as a group have been studied since the 1950s but only recently has angelicin begun to come into its own as the subject of several biological studies. Angelicin has demonstrated anti-cancer properties against multiple cell lines, exerting effects *via* both the intrinsic and extrinsic apoptotic pathways, and also demonstrated an ability to inhibit tubulin polymerization to a higher degree than psoralen. Besides that, angelicin too demonstrated anti-inflammatory activity in inflammatory-related respiratory and neurodegenerative ailments *via* the activation of NF-κB pathway. Angelicin also showed pro-osteogenesis and pro-chondrogenic effects on osteoblasts and pre-chondrocytes respectively. The elevated expression of pro-osteogenic and chondrogenic markers and activation of TGF-β/BMP, Wnt/β-catenin pathway confirms the positive effect of angelicin bone remodeling. Angelicin also increased the expression of estrogen receptor alpha (ERα) in osteogenesis. Other bioactivities, such as anti-viral and erythroid differentiating properties of angelicin, were also reported by several researchers with the latter even displaying an even greater aptitude as compared to the commonly prescribed drug, hydroxyurea, which is currently on the market. Apart from that, recently, a new application for angelicin against periodontitis had been studied, where reduction of bone loss was indirectly caused by its anti-microbial properties. All in all, angelicin appears to be a promising compound for further studies especially on its mechanism and application in therapies for a multitude of common and debilitating ailments such as sickle cell anaemia, osteoporosis, cancer, and neurodegeneration. Future research on the drug delivery of angelicin in cancer, inflammation and erythroid differentiation models would aid in improving the bioproperties of angelicin and efficacy of delivery to the targeted site. More in-depth studies of angelicin on bone remodeling, the pro-osteogenic effect of angelicin in various bone disease models and the anti-viral implications of angelicin in periodontitis should be researched. Finally, studies on the binding of angelicin toward regulatory genes, transcription factors, and receptors can be done through experimental research supplemented with molecular docking and molecular dynamics simulation.

## Introduction

The use of plants as traditional medicine is common and has prevailed in many different cultures over time. Despite a lack of formal scientific evidence, the belief in the knowledge, traditions, and religious practices that endorse this is strong enough to have sustained this practice over the generations. It seems unlikely that these practices would have persisted for so long in the complete absence of beneficial effects. This suggests plants already used in traditional medicine represent an excellent start point in research to discover new, effective drugs to treat various human illnesses including cancer, bacterial infections, and cardiovascular disease, to name a few. The market trends which have shifted toward a demand for greener, cost-saving, and sustainable sources also created a drive to pursue plant bioprospecting; plants are easily obtained from the environment and are therefore regarded as a cheaper and safer source for consumers (Chandra, 2014).

**Figure 1:**
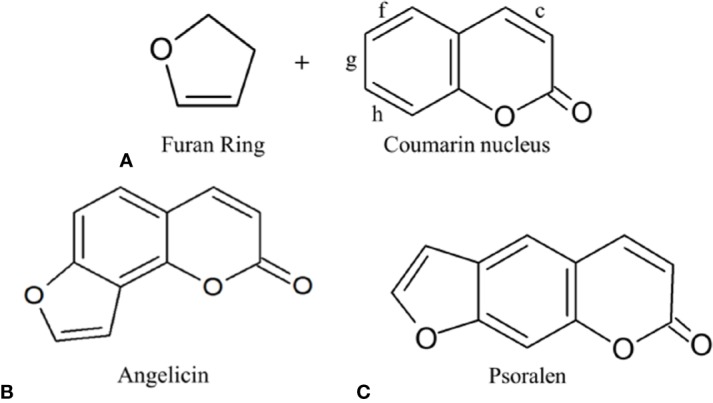
**(A)** Furocoumarin's several different possible attachments of the furan ring on the coumarin nucleus; **(B)** Angular furocoumarin: Angelicin; **(C)** Linear furocoumarin: Psoralen.

Among the compounds that have emerged from plant bioprospecting studies are the furocoumarin family of compounds that have been researched since the 1950s (Bordin et al., 1991). They are a family of natural active compounds that can be found in many different plants, vegetables and fruits we consume like parsnips, celery, figs, etc (Chaudhary et al., 1985; Lohman and McConnaughay, 1998; Marrelli et al., 2012). These compounds are mainly produced under stressful conditions as a self-defence mechanism against insect predation, fungal invasion, and bacterial attack. For example, celery infected with the fungus *Sclerotinia sclerotiorum* before storage has been found to have increased expression of furocoumarins—such as psoralen, 5-methoxypsoralen (5-MOP) and 8-methoxypsoralen (8-MOP)—as a response toward the fungal invasion (Chaudhary et al., 1985). Other studies showed that furocoumarins also displayed anti-feeding properties against insects and inhibition toward bacterial invasion (Yajima and Munakata, 1979; Berenbaum et al., 1991; Raja et al., 2011). The main skeletal structure of furocoumarin compounds encompasses a coumarin unit fused with a furan ring. The varying derivatives of furocoumarins can be formed by the fusing of the furan ring in either 2, 3- or 3,2- arrangements on the c, f, g, or h bonds of the coumarin unit as can be seen in **Figure 1** (Santana et al., 2004; Kitamura and Otsubo, 2012). Among the many isomeric derivatives of the furocoumarins, the most commonly reported furocoumarins isomeric forms are linear and angular furocoumarins, with psoralen and angelicin being the most well-known of its isomers respectively (Munakata et al., 2016).

**Figure 2:**
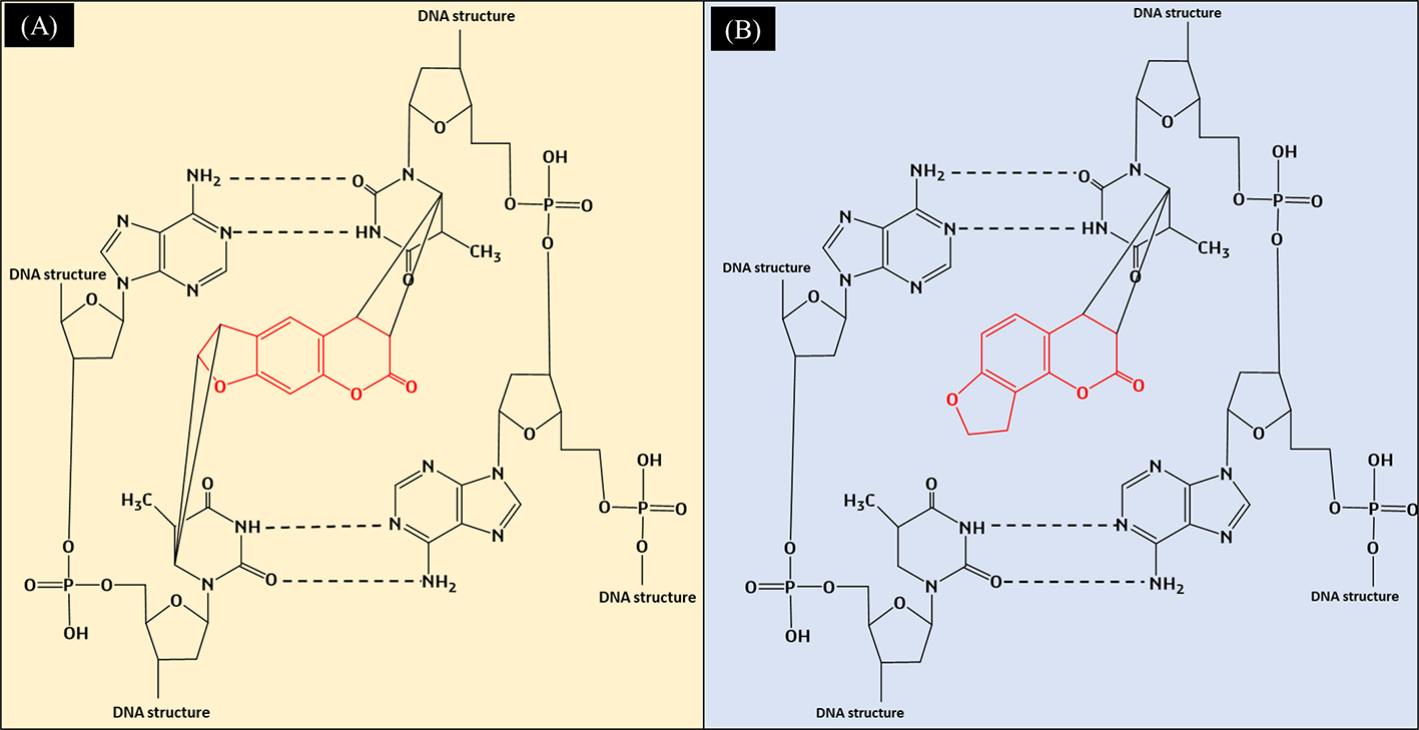
**(A)** Formation of interstrand crosslinks in the DNA by psoralen when exposed to UVR; **(B)** Formation of monoadducts in the DNA by angelicin when exposed to UVR.

Both angelicin and psoralen are photosensitizing furocoumarins, but they interact very differently in the presence of ultraviolet radiation (UVR). With its linear structure, psoralen was discovered to form both monoadducts as well as interstrand cross-links with DNA when irradiated with UVR. When irradiated, the 3',4' or 4',5' double bonds of the molecule will covalently bind with the 5',6'-double bond of the pyrimidines from both sides of the DNA strand by absorbing photons, leading to the crosslinks that can be seen in **Figure 2** (Ben-Hur and Elkind, 1973; Nagy et al., 2010). Angelicin, on the other hand, can only form monoadducts due to its steric structure (Grant et al., 1979). The monoadducts formed on DNA by angelicin are also quick to be repaired by the cells and hence, angelicin imposes lower phototoxicity as compared to psoralen (Bordin et al., 1976; Joshi and Pathak, 1983). Although both compounds are photosensitizing compounds, psoralen was favored to be used in phototherapy as it produces desirable effects such as the reduction of lesions in vitiligo, psoriasis, atopic dermatitis, etc. (Vallat et al., 1994; Lee and Jang, 1998; Petering et al., 2004). However, usage of psoralen has reduced, with other modalities of phototherapy treatments and alternative drugs being preferred as psoralen was found to increase the risk of skin cancer and other systemic side effects when consumed (Llins and Gers, 1992; Stern et al., 1997).

**Figure 3:**
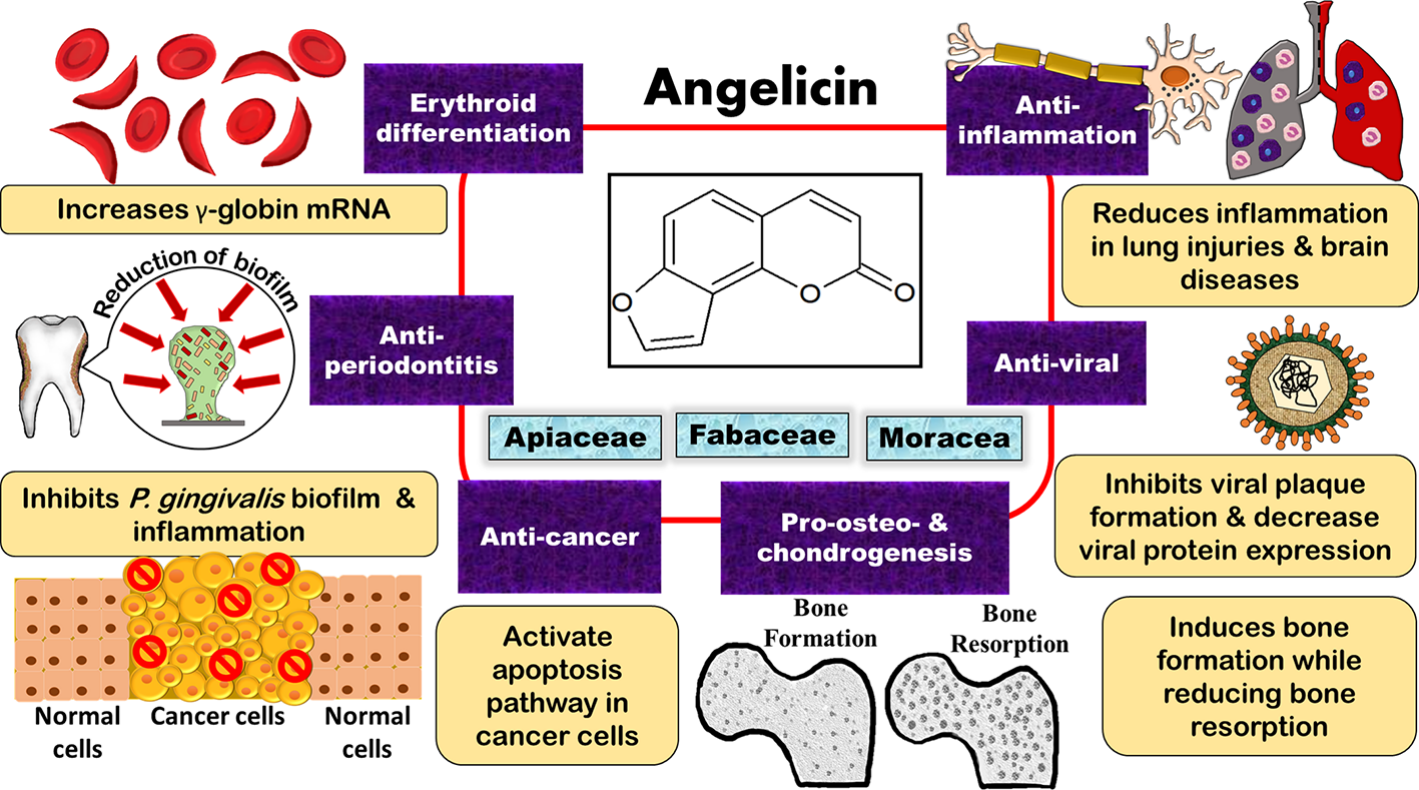
Potential bioproperties of the furocoumarin, angelicin, as an anti-cancer, anti-inflammation, anti-viral, anti-periodontitis, erythroid differentiating, and pro-osteo- and chondrogenic therapeutic agent.

Though psoralen has been the subject of many studies due to its phototherapeutic properties, angelicin too has demonstrated multiple effects including anti-cancer, anti-viral, anti-inflammatory, anti-microbial, pro-osteogenic and chondrogenic differentiation, and erythroid differentiating properties (Lampronti et al., 2003; Cho et al., 2013; Liu et al., 2013; Wang et al., 2017a; Li et al., 2018). Regarding its anti-viral and erythroid differentiating properties, other studies have also compared angelicin to the common drugs on the market, such as ganciclovir (GCV) and hydroxyurea, and it had was shown that angelicin had almost equal or even better effect as compared to these drugs (Lampronti et al., 2003; Cho et al., 2013). This review evaluates and summarises the findings on the bioactivities of angelicin to showcase the potential of angelicin to be used as a therapeutic agent. A summary of all the biological properties of angelicin that were reported can also be seen in **Table 1** and **Figure 3**.

**Table 1:** Bioactivities of angelicin reported in *in vitro* and *in vivo* experimental models.

Bioactivity	Experimental model	Exposure time	Chosen Concentration	Efficacy	References
Anti-cancer	SH-SY5Y neuroblastoma cell line	48 h *in vitro* assay	0, 10, 30, 50, 70 and 100 μM angelicin	(a) Cell viability IC_50_: 49.56μM (b) Significant fold decrease of BcL-2, Bcl-xL, and Mcl-1: **≥** 30 μM (c) Significant fold increase of cleaved caspase 9: ≥50 μM (d) Significant fold increase of cleaved caspase 3: **≥**40 μM (e) Decrease in procaspase 9 expression (f) No changes were seen on PI3K/AKT/GSK-3β, MAPK and Fas/FasL signaling pathways.	(Rahman et al., 2012)
	HL-60 cell line	1. 24 h *in vitro* assay 2. 48 h *in vitro* assay	40 and 80 μg/ml angelicin	(a) Cell viability IC_50_ at 24 and 48 h: 1) 148.4 μg/ml 2) 41.7 μg/ml (b) Dose-dependent upregulation of Bax and downregulation of Bcl-2 when treated with angelicin at both 24 and 48 h	(Yuan et al., 2015)
	1. Human lung carcinoma A459 cell line. 2. Female nude mice (aged 5 weeks	1. 24 h for *in vitro* assay 2. *In vivo* experiments: (a) Oral gavage 100 mg/kg for 4 consecutive weeks. (b) Angelicin (100 mg/kg/day) was fed through intragastric administration daily for 4 weeks.	*In vitro* assay: 10, 25, 50μM angelicin *In vivo* assay: 1. Oral gavage 100 mg/kg 2. Intragastric administration 100 mg/kg/day	*1. In vitro* assays: (a) Cell viability IC_50_: 50.14 μM (b) Significant apoptosis rate using Annexin V-FITC and TUNEL positive cells: ≥ 25 μM (c) Significant increase in Bax/BCL-2 ratio: ≥ 10 μM (d) Significant increase in cleaved caspase 3 and 9: ≥ 10 μM (e) significant cell count decrease in G1 and an increase in G2/M phase: ≥ 25 μM (d) Significant decrease in cyclin B1: **≥** 10 μM (e) Significant decrease in cyclin E1 and cdc2: **≥** 25 μM (f) Significant inhibition of cell migration, adhesion, and invasion: **≥** 25 μM (g) Significant increase in E-cadherin: ≥ 10 μM (h) Significant decrease in MMP2 and MMP9: ≥25 μM (i) Significant increase in pERK/ERK and pJNK/JNK: ≥ 10 μM (f) No changes were seen on p38 MAPK and AKT 2. *In vivo* assay (a) Significant decrease in tumor size and weight compared to the control. (b) Significant decrease in the number of lung lesions compared to the control. Significant increase in the ratio of TUNEL-positive cells. A decrease in the expression levels of MMP2 and MMP9 and increased expression of E-cadherin in the immunohistochemistry assay.	(Li et al., 2016)
	1. HepG2 hepatoblastoma cell line 2. Huh-7 hepatocellular carcinoma cell line 3. Male BALB/c-nu/nu mice (aged 4–6 weeks, mean weight= 25 g)	1. 48 h for *in vitro* assays except in flow cytometry and TUNEL assay, Huh-7 were incubated for 36 h with angelicin. 2. *In vivo* experiments: Intraperitoneal injection of angelicin daily.	*In vitro* assay: 0, 10, 30, and 60 μM angelicin *In vivo* assay: 20 and 50 mg/kg angelicin	*In vitro* assay 1. HepG2 cell line (a) Cell viability IC_50_ for HepG2: 90 ± 6.565 μM (b) Significant decrease of PI3K/GADPH: ≥ 90 μM (c) Significant decrease of p-AKT/AKT: ≥ 60 μM (d) Upregulation of cleaved caspase 3, caspase 9, Bax, and cytochrome c (e) Downregulation of Bcl-2 2. Huh-7 cell line (a) Cell viability IC_50_ for Huh-7: 60 ± 4.256 μM (b) Significant decrease of PI3K/GADPH: **≥** 30 μM (c) Significant decrease in p-AKT/AKT: ≥ 10 μM (d) Upregulation of cleaved caspase 3, caspase 9, Bax, and cytochrome c (e) Downregulation of Bcl-2 *In vivo* assay (a) Significant decrease in tumor weight: ≥ 50 mg/kg (b) Significant increase of TUNEL-positive cells: ≥ 20 mg/kg (C) Significant decrease in Ki-67 values: **≥** 50 mg/kg (d) Significant decrease in p-VEGFR2 values: ≥ 50 mg/kg	(Wang et al., 2017a)
	Cancer cell lines 1.Human renal carcinoma (Caki) cell line 2. Hepatocellular carcinoma (Sk-hep1) cell line 3. MDA-MB-361 cell line Normal cell lines: 1. Mouse renal tubular epithelial (TCMK-1) cell line 2. human skin fibroblasts (HSFs) cell line	24 h *in vitro* assay	1) 100 μM angelicin with 50 ng/ml of TRAIL 2) 50, 75, 100 μM angelicin alone 3) 50 ng/ml TRAIL alone	Combination of angelicin and TRAIL (a) Significant increase in the percentage of sub G1 population on Caki cells: ≥ 50 μM angelicin + 50 ng/ml TRAIL (b) Increase in cleaved PARP: 100 μM angelicin + 50ng/ml TRAIL (c) Increased in caspase 3 activity:100 μM angelicin + 50ng/ml TRAIL (e) Down-regulation of c-FLIP:100 μM angelicin + 50 ng/ml TRAIL (f) No changes were seen in apoptosis related proteins: cIAP1, XIAP, DR5, Mcl-1, Bcl-2, Bcl-xL, and survivin. Angelicin alone increases Bim expression but combination treatment attenuated the effect. (g) Cytotoxicity of combination treatment of angelicin and TRAIL was independent of ER stress and ROS signaling (h) Combination treatment of angelicin and TRAIL did not affect normal cells but causes apoptosis in sk-hep1, MDA-MB-361 cells	(Min et al., 2018)
	Human prostate cancer (PC-3) cell line	48 h *in vitro* assay	0, 25, 50, and 100 μM angelicin	(a)Cell viability IC_50_: 65.2 μM	(Wang et al., 2015a)
	1. Human hepatocellular carcinoma (HepG2) cell line 2. Human epithelioma (Hep2) cell line 3. Colorectal carcinoma (HCT116) cell line 4. rhabdomyosarcoma (RD) cell line 5. Human breast adenocarcinoma (MCF7) cell line 6. normal cell line human lung fibroblasts (WI-38) cell line			(a)Cell viability IC_50_ and IC_50_ of WI-38/IC50 (SI) 1. HePG2 cell line: 13.8 ± 0.64 μM; SI: 7.4 (angelicin) • Psoralen: 17.2 ± 0.21 μM; SI: 5.6 2. Hep2 cell line: 11.5 ± 2.28μM; SI: 8.9 (angelicin) • Psoralen: 20.5 ± 0.01 μM; SI: 4.7 3. HCT116 cell line: 8.7 ± 0.72 μM; SI: 11.8 (angelicin) • Psoralen: 26.1 ± 0.51 μM; SI: 3.7 4. RD cell line: 12.9 ± 1.33 μM; SI: 7.9 (angelicin) • Psoralen: 28.3 ± 0.22 μM; SI: 3.4 5.MCF7 cell line: 7 ± 0.91 μM; SI: 14.7 (angelicin) • Psoralen: 32.4 ± 0.43 μM; SI: 3 6. WI-38 cell line: 102.9 ± 1.35 μM (angelicin) • Psoralen: 97.2 ± 0.92 μM (b) Tubulin polymerization inhibition by angelicin: 57.1 ± 6.3% (Half-maximal inhibitory concentration of tubulin inhibition: 7.92 ± 1.9 μM) • Tubulin polymerization inhibition by psoralen: 30.13 ± 6.13% (c) Rank score match of angelicin with colchicine binding residues on tubulin microtubules: -53.19 ± 0.88 kcal/mol • Psoralen: -45.49 ± 0.46 kcal/mol (d) Inhibition of histone deacetylase 8 inhibitory (HDAC8) assay by angelicin: 33.86 ± 2.86% • Psoralen: 30.70± 3.52% (e) Angelicin rank score match with the binding site of HDAC8: -77.23 ± 0.73 kcal/mol • Psoralen: -72.75 ± 1.82 kcal/mol	(Mira and Shimizu, 2015)
	1. Human myelogenous leukemia (K562) cell line 2. Human myelogenous leukemia with multidrug resistance (K562/A02) cell line	48 h *in vitro* assay	For cytotoxicity experiments, different concentrations of compounds 1–9 were added into designated wells, and for MDR reversal experiments, different concentrations of doxorubicin (DOX) were added into designated wells with or without compounds 1–9 (10 mmol/L).	(a) Cell viability IC_50_: 1. K562 cell line: 0.41 ± 0.20 μmol/L 2. K562/A02 cell line: 42.7 ± 0.14 μmol/L (b) reversal fold (RF) on K562/A02 =IC_50_ of cytotoxic DOX alone/IC_50_ of cytotoxic DOX: 0.97	(Wang et al., 2016)
	The marrow cavity of cortical bone (tuberosity region of the lower part of the tibia) of nude rats was injected with UMR-106 cells (rat osteosarcoma)	The treatment was administered *via* intratumoral multi-point injection when the tumor is 0.5 cm x 0.5 cm large. All treatment except cisplatin was administered for 2 courses (5 days per course) with a day break in between two courses. For the cisplatin treatment, the rats were only treated on day 1, 4, 7, and 10.	1. Psoralen: 320 μg/(kg.d) and 1,600 μg/(kg.d) 2. Angelicin: 320μg/(kg.d) and 1,600 μg/(kg.d) 3. Cisplatin: 2 mg/kg	1. No significant reduction in body weight was seen for both psoralen and angelicin treatment. However, the bodyweight of those treated with cisplatin experienced significant body weight loss after the 3rd of administration. 2. After the 3rd day administering psoralen and angelicin, the rats showed some toxic reactions such as lassitude, hypoactivity, and writhing movements. No writhing movements were seen in the treatment of cisplatin. 3. The osteosarcoma volume and weight were significantly decreased in all treatments. 4. Significant decrease in serum ALP in all treatments. 5. All treatment showed a decrease in tumor cell density, increased amount of necrotic tumor cells, cell debris, and protein-like substances can be found in the center, shrinking of cells with nuclei condensation, decreased mitosis. Some tumor tissue experienced haemorrhaging, increased intercellular substances and infiltration of lymphocyte. No metastatic lesion in the abdomen or changes in the liver, spleen, kidney, hearts, and lungs. 6. The higher doses of psoralen and angelicin, together with cisplatin, displayed inflammatory cell aggregation and tubulointerstitial vascular dilation and congestion in the renal intercellular space. 7. Electron microscope: (a) Low doses of angelicin and psoralen: Cells were arranged loosely with dilated endoplasmic reticulum, damaged and dissolved nuclear membranes, condensed nuclei, large vacuoles in cytoplasm, chromatins distributed in clumps, rare sighting of organelles and typical necrotic features. (b) High doses of angelicin and psoralen: Ruptured cells, loose and disappeared cytoplasm, ruptured and rough nuclear membranes, blurred intranuclear structure, loose chromatins, and large mitochondria. (c) Cisplatin: Cavitated and denatured mitochondria and matrices, homogenized nuclei, deepened colour, clumped chromosomes, and flocculent shaped lipid droplets. 8. No significant difference in the number of peripheral red blood cells, white blood cells, platelets, haemoglobin and bone marrow nucleated cells between the control group and psoralen and angelicin treated group. Cisplatin had a significant decrease in white blood cells and bone marrow nucleated cells.	(Lu et al., 2014)
	1. Human cervical carcinoma cell line (HeLa) 2. Cervical squamous cell carcinoma cell line (SiHa) 3. Nontumor cervical epithelial cell line (ECT1/E6E7)	1. Cell viability assay: Cells were exposed to angelicin for 24 h. 2. Cell viability assay (IC_30_): Cells were exposed to angelicin at their IC_30_ for 5 days. 3. Cell cycle, cell proliferation, colony formation, tumor formation, migration and invasion assays: Exposure time to angelicin were not mentioned 4. Apoptosis and autophagy assay: Cells were exposed to angelicin for 24 h.	1. Cell viability assay to determine the IC_30_ and IC_50_ for each cell line: 40, 60, 80, 100, 120, 140, 160, 180, or 200 µM angelicin 2. The following assays (5 days cell viability, proliferation, cell cycle, colony formation, tumor formation, migration, invasion and autophagy assays) were demonstrated using the IC_30_ angelicin concentration of each cell line. 3. The following assays (apoptosis assay) were demonstrated using IC_50_ angelicin concentration for each cell line.	1. Measurement of 30% and 50% inhibitory concentration (IC_30_ and IC_50_) after treatment with angelicin for 24 h: (a) HeLa cells: IC_30__=_ 27.8μM; IC_50__=_ 38.2 μM (b) SiHa cells: IC_30__=_ 36.6μM; IC_50__=_ 51.3 μM (c) ECT1/E6E7 cells: IC_30__=_ 82.7 μM; IC_50__=_ 138.5μM 2. Cell viability of cells after incubating at their IC_30_ concentration: (a) HeLa cells: ≥ day 4 (b) SiHa cells: ≥ day 4 2. Angelicin decreased the proliferation of both HeLa and SiHa cells 2. Cell cycle assay: Both HeLa and SiHa cells were significantly arrested at the G1/G0 phase and significantly decreased at G2/M 3. Angelicin decreased the colony formation, tumor formation, migration and invasion of both HeLa and SiHa cells 4. Angelicin significantly increase apoptotic death rate in both HeLa and SiHa cell lines 5. Autophagy assay: (a) Angelicin increased the accumulation of LC3B in the cytoplasm of both HeLa and SiHa cell lines (b) Angelicin decreased the LC3B and LC3B-II quantity in both HeLa and SiHa cell lines (c) Angelicin decreased Atg3, Atg7, Atg12-5, and LC3B protein expression in both HeLa and SiHa cell lines. 6. Angelicin increased the phosphorylation of mTOR protein and decreased the protein level of LC3B-II in both cell lines.	(Wang et al., 2019b)
	1. Docking of angelicin to ERα, progesterone receptor (PR), epidermal growth factor receptor (EGFR) and mTOR 2. MCF-7 cells	1. ERα reporter antagonist assay: 22–24 h treatment with angelicin 2. mTOR inhibition assay: MCF-7 cells were treated with angelicin for 24 h	Not specified	1. The binding energy of angelicin: (a) ERα: -12.01 kcal/mol (b) PR: -11.63 kcal/mol (c) EGFR: -12.60 kcal/mol (d) mTOR: -13.64 kcal/mol 2. Docking score (Ref/selected bio-molecules) and nature of interaction of angelicin: (a) ERα: -34.44/-12.01 kcal/mol, hydrophobic and polar H interactions (b) PR: -21.11/-11.63 kcal/mol, hydrophobic and polar H interactions (c) EGFR: -19.22/-12.60 kcal/mol, hydrophobic and polar H interactions (d) mTOR: -46.09/-13.64 kcal/mol, hydrophobic and polar H interactions 3. IC50 of angelicin in reducing luminescence intensity (antagonizing ERα): 11.02 µM 4. Angelicin attenuates the upregulated EGFR expression in MCF-07 cells 5. Angelicin was unable to inhibit mTOR	(Acharya et al., 2019)
Anti-inflammatory	1. RAW 264.7 mouse macrophage cell line 2. BALB/c male mice (8-10 weeks old with the weight of 18-20g each)	(1) *In vitro* assay: 1 h pretreatment with angelicin followed by 18 h of 4 µg/ml LPS stimulation (2) *In vivo* assay: 1 h pretreatment with angelicin intraperitoneally followed by 10µg LPS stimulation. Mice were sacrificed after 6 h. The lungs were lavaged and excised.	1) 0–200 µg/ml angelicin for *in vitro* assay 2) 1, 5, and 10 mg/kg angelicin for *in vivo* assay	(a) *In vitro* assays: 1. Concentrations of 100 and 200 µM were able to reduce cell viability 2. Significant decrease of TNFα and IL-6: ≥ 12.5 µg/ml (b) *In vivo* assays: 1. Significant decrease in total cells and neutrophils in bronchoalveolar lavage fluid (BALF): ≥ 1 mg/kg 2. Significant decrease in macrophages in BALF: ≥ 5 mg/kg 3. Significant decrease in TNF-α and IL-6 in BALF: ≥ 1mg/kg 4. Significant decrease in lung injury score: ≥ 1 mg/kg 5. Significant decrease in lung W/D ratio: ≥ 1 mg/kg 6. Significant decrease in MPO activity: ≥ 1 mg/kg 7. Significant decrease in p-p65NF-κB -actin ratio when induced with 500 µg/kg LPS: ≥ 1mg/kg 8. Significant decrease in p-IκBα/β- actin ratio when induced with 500 µg/kg LPS: ≥ 1 mg/kg 9. Significant increase in IκBα/β- actin ratio when induced with 500 µg/kg LPS: ≥ 1 mg/kg 10. Significant decrease in pJNK/JNK ratio and p-p48/p38 ratio when induced with 500 µg/kg LPS: ≥ 1 mg/kg	(Liu et al., 2013)
	1. Female BALB/c mice with weight of 18-20g each	*In vivo* assay: Angelicin was given 1 h as a pretreatment before OVA treatment after initial sensitization. After 24 h, the mice were sacrificed.	2.5, 5, 10 mg/kg angelicin	1. Significant decrease of total inflammatory cell count, eosinophils, neutrophils, lymphocytes, and macrophages: ≥ 2.5 mg/kg 2. Significant decrease of IL-4, IL-5, and IL-13 in BALF: ≥ 2.5 mg/kg 3. Significant decrease in IgE production: ≥ 2.5 mg/kg 4. Significant alleviation of OVA-induced airway hyperresponsiveness (expressed at enhanced minute pause (Penh) that reflects pulmonary resistance when treated with 10 mg/kg methchacholine: ≥ 2.5 mg/kg 5. Significant decrease in p-p65/β-actin ratio and p-IκB/β-actin ratio: ≥ 2.5 mg/kg	(Wei et al., 2016)
	1. Mouse microglia BV-2 cell line which is generated through infection of primary microglial cell with vraf/v-myc oncogene carrying retovirus. 2. Mouse hippocampal HT22 cell line	1. Measurement of NO in BV-2 cells: 2 h pre-treatment with compound 2. Measurement of neuroprotective effect against hydrogen peroxide (H_2_O_2_) in HT22 cells: 6 h co-treatment with H_2_O_2_	25, 50, 100 µM angelicin	1. Significant decrease of LPS induced NO in BV-2 cells by angelicin: ≥ 50 µM • No significant change of LPS induced NO in BV-2 cells by psoralen 2. Significant (p < 0.01) increase in cell viability in HT22 cells treated with H_2_O_2_ by angelicin: ≥25 µM • Significant (p < 0.05) increase in cell viability in HT22 cells treated with H_2_O_2_ by psoralen: 25 µM (higher concentrations showed no significant changes in cell viability as compared to treating the cells alone with H_2_O_2_)	(Kim et al., 2016)
	1. Human neutrophils obtained from the venous blood of healthy adult patients (age: 20-28 years old) 2. RAW264.7 murine macrophage cell line	1. Measurement of the generation of superoxide anion and release of elastase: 5 min of incubation with the compounds obtained from *Psoralea corylifolia* L. 2. To determine NO production: 24 h of incubation with the compounds obtained from *Psoralea corylifolia* L.	1. Measurement of superoxide anion generation: 30–0.01 µM of compounds obtained from *Psoralea corylifolia* L. 2. Determination of NO production: 0,3, 15, 30, and 60 µM of compounds obtained from *Psoralea corylifolia* L.	1. Percentage of angelicin induced inhibition of superoxide anion at 30 µM: 44.91± 6.46% (Significant against positive control) • IC_50_ of psoralen induced inhibition of superoxide anion: 5.91± 3.22 µM (Significant against positive control) 2. Percentage of angelicin induced inhibition of elastase release at 30 µM: 27.73 ± 4.22 (Significant against positive control) • Percentage of psoralen induced inhibition of elastase release at 30µM: 24.70 ± 5.99 (Significant against positive control) 3. IC_50_ inhibition against the formation of NO by RAW264.7 murine macrophages by angelicin: 56.82 ± 3.7 µM (Significant against positive control) • IC_50_ inhibition against the formation of nitric oxide by RAW264.7 murine macrophages by psoralen: 40.15 ± 2.27 µM (Significant against positive control)	(Chen et al., 2017)
Pro-osteogenesis	Primary rat calvarial osteoblasts isolated from calvaria of newborn Winstar rats	1. Cell proliferation assay: the cells were treated with angelicin for 24 and 48 h 2. ALP assay: the cells were treated with angelicin for 24, 48 and 72 h	1, 10, 100 µM angelicin	1. No changes in proliferation rate or cell toxicity were seen. 2. No changes in ALP activity was seen	(Li et al., 2014)
	1. Murine pre-osteoblast (MC3T3-E1) cells 2. HEK293T cells transfected with (CAGA) 12-Luc-reporter plasmid and internal control (pRL-TK vector)	1. Cell viability assay on MC3T3-E1 cells: 72 h incubation with angelicin 2. Measurement of the luciferase activity (activation of TGF-β1 reporter gene): HEK293T cells were treated with angelicin for 12 h. 3. Measurement of mRNA and protein expression level of M3T3-E1 cells after incubation of cells with angelicin for 72 h. Before treatment, 24 h synchronization was done to cause the cells to quiescence at G0 stage.	1. Cell viability assay, measurement of mRNA and protein expression level on MC3T3-E1 cells: 0.1, 0.01, 0.001 and 0.0001µM angelicin 2. Measurement of the luciferase activity (activation of TGF-β1 reporter gene) in HEK293T cells: 0.1, 0.01, 0.001 and 0.0001 µM	1. Significant increase in MC3T3-E1 cell viability: ≥ 0.01 μM angelicin 2. Significant increase in relative luciferase activity which indicates the of TGF-β1 reporter gene activity: ≥ 0.01 μM angelicin 3. Significant increase in Type I collagen mRNA levels: ≥ 0.01 μM angelicin 4. Significant decrease in Smad7 protein levels: ≥ 0.01 μM angelicin	(Zhang and Ta, 2017)
	OB-6 osteoblastic cells	Treatment of cells with angelicin, H_2_O_2_ or combination of both for 12, 24 and 48 h for all assays except when measuring the protein expression of β-catenin, tankyrase, and Wnt. The exposure time of cells to angelicin and/or H_2_O_2_ was not mentioned for protein expression analysis.	There are 4 groups: 1. Control group 2. Angelicin only group: cells treated with 1µM angelicin 3. H_2_O_2_ only group: cells treated with 100µM H_2_O_2_ 4. Combined group: cells treated with both 1µM angelicin + 100µM H_2_O_2_	1. Combined group significantly increase the percentage of cell viability as compared with H_2_O_2_ only group: ≥ 12 h treatment 2. Combined group significantly decrease the apoptotic rate as compared with H_2_O_2_ only group: ≥ 12 h treatment 3. Combined group significantly decrease the ROS levels as compared with H_2_O_2_ only group ≥ 12 h treatment 4. Combined group significantly increase complex I activity as compared with H_2_O_2_ only group: ≥ 12 h treatment 5. Combined group significantly increase the calcium levels as compared with H_2_O_2_ only group: ≥ 12 h treatment 6. Combined group significantly increase the OCN and RUNX2 mRNA expression as compared with H_2_O_2_ only group: ≥ 12 h treatment 7. Combined group significantly increase the β-catenin and tankyrase protein expression as compared with the H_2_O_2_ only group. The expression of Wnt protein experienced no changes with any treatment.	(Li et al., 2019)
	Primary rat osteoblasts cells obtained from female Winstar rat with a weight of 5-6g	1. Cell proliferation assay: 24, 48, 72 h incubation with angelicin 2. Measurement of ALP activity: 7 days incubation with angelicin 3. Quantification on level of osteoblast mineralization: 12 days incubation with angelicin 4. Gene and protein expression analysis: 24 h incubation with angelicin	0.1, 1, 10µM angelicin	1. Significant increase in osteoblasts cell proliferation after 48 and 72 h incubation with ≥ 1µM angelicin and ≥ 0.1 µM angelicin respectively. 2. Significant increase in ALP relative activity in osteoblasts after 7 days incubation with angelicin: ≥ 0.1 µM 3. Significant increase in mineralization of osteoblasts after 12 days incubation with angelicin: ≥ 1 µM 4. Significant increase in ALP and OCN mRNA after 24 h incubation with angelicin: ≥ 1 µM 5. Significant increase in RUNX2 and COL1A1 mRNA after 24 h incubation with angelicin: ≥ 0.1 µM 6. Significant increase in RUNX2, BMP2, and ERα protein expression after 24 h incubation with angelicin: ≥ 1 µM 7. Significant increase in β-catenin protein expression after 24 h incubation with angelicin: ≥ 0.1 µM	(Ge et al., 2019)
	1. Primary femoral BMSCs obtained from C57BL/6 mice (4 weeks old) 2. Female C57BL/6 mice (aged 2 months)	(a) *In vitro* assay: 1. Measurement of cell growth after 2 days of treatment with angelicin 2. Measurement of ALP- positive cells after 14 days of treatment with angelicin 3. Analysis of the adipogenesis rate after adipogenic differentiation at day 7 and day 14 4. Measurement of OCN and RUNX2 expression in BMSC differentiated osteoblasts after 14 days of treatment (Immunocytochemistry and western blotting) 5. Measurement of C/EBPβ and PPARγ expression in BMSC differentiated adipocytes after 7 and 14 days of treatment (Immunocytochemistry and western blotting) 6. Assessment of the changes in the mTORC1 signaling pathway (4E/BP1 and p-S6) in BMSC adipogenesis after treatment with angelicin for 7 and 14 days. (b) *In vivo* assay: The mice were administered angelicin for 5 days prior to overiectomy and angelicin was continuously administered for 2 months after the operation. 1. Measurement of RUNX2 and PPARγ expression in the distal femur 2. Measurement of the number of adipocytes in the bone marrow 3. Measurement of the trabecular thickness (Tb.Th), bone volume/total volume (BV/TV) and trabecular number (Tb.N) in mouse distal femur	(a) *In vitro* assay: 1. Measurement of cell growth: 0.1, 1, 10, 100, and 1,000 μM angelicin 2. Other assays: 5, 10 and 20μM angelicin (b) *In vivo* assay: 20mg/kg angelicin	(a) *In vitro* assay: 1. Cell growth: No changes in the cell growth up to 100 μM angelicin. Concentrations of 1,000 μM significantly decrease cell viability. 2. Significant increase in ALP- positive cells: ≥ 5 μM angelicin 3. Significant decrease in adipogenesis rate after adipogenic differentiation at day 7 and day 14: ≥ 5 μM angelicin 4. Significant increase in OCN and RUNX2 expression in BMSC differentiated osteoblasts after 14 days of treatment (Immunocytochemistry and western blotting): ≥ 5 μM angelicin 5. Significant decrease in C/EBPβ and PPARγ expression in BMSC differentiated adipocytes after 7 and 14 days of treatment (Immunocytochemistry and western blotting):≥ 5 μM angelicin 6. Significant increase and decrease in 4E/BP1 and p-S6 expression respectively in BMSC adipogenesis after treatment with angelicin for 7 and 14 days: ≥ 5 μM angelicin 7. There was an increase and decrease in 4E/BP1 and p-S6 expression respectively in BMSC osteogenic differentiation after treatment with angelicin for 14 days (b) *In vivo* assay: 1. Significant increase and decrease in RUNX2 and PPARγ expression respectively in the distal femur after treatment with angelicin 2. Significant decrease in the number of adipocytes in the bone marrow after treatment with angelicin 3. Significant increase in the trabecular thickness (Tb.Th), bone volume/total volume (BV/TV) and trabecular number (Tb.N) in mouse distal femur after treatment with angelicin	(Wang et al., 2017b)
	1. MC3T3-E1 cells 2. C57BL/c mice (8 weeks old)	(a) *In vitro* assay: 1. Quantification of MC3T3 cell mineralization: 14 days incubation with angelicin 2. Measurement of relative ALP activity: 9 days incubation with angelicin 3. Gene expression analysis: 24 and 72 h incubation with angelicin 4. Analysis of AhR protein expression in nucleus and cytoplasm of osteoblasts: 24 h incubation with angelicin 5. Analysis of the binding affinity of angelicin to AhR using drug affinity responsive target stability (DARTS) analysis. MC3T3-E1 cell lysate (5 mg/ml) was incubated with 10µM angelicin for 1 h at room temperature before digestion of protein with pronase to 1:1,000 or 1:2,000 for 30 min. 6. Analysis of ERα protein expression: 5 days incubation with angelicin (b) I*n vivo* assay: 1. Analysis of CYP1A1 content in serum after injection of mice with angelicin	(a) *In vitro* assays: 2, 10, and 50 µM angelicin (b) *In vivo* assays: 10 mg/kg angelicin	(a)*In vitro* assay 1. Significant increase in MC3T3-E1 mineralization after 14 days incubation with angelicin: ≥ 10 μM 2. Significant increase in relative ALP activity after 9 days incubation with angelicin: ≥ 10 μM 3. Significant increase in ALP mRNA after 24 and 72 h incubation with ≥ 10 μM and ≥ 2 μM angelicin respectively. 4. Significant increase in COL1A1 mRNA after 24 and 72 h incubation with ≥ 10 μM and ≥ 2 μM angelicin, respectively. 5. Significant increase in RUNX2 mRNA after 24 and 72 h incubation with ≥ 50 μM and ≥ 10 μM angelicin respectively. 6. Significant decrease in CYP1A1 mRNA after 24 and 72 h incubation with ≥ 2 μM angelicin. 7. Significant increase in AhR protein levels in the cell cytoplasm while a significant decrease in AhR protein levels in the cell nucleus after incubation with ≥ 2 μM angelicin for 24 h 8. Significant binding of 10 µM angelicin to AhR, inhibiting proteolysis at 1:1,000 and 1:2,000 protein ratios. 9. Significant increase in ERα protein expression after 5 days incubation with angelicin: ≥ 2 μM (b) *In vivo* analysis: 1. Significant decrease in CYP1A1 content in serum after treatment of mice with 10 mg/kg angelicin	(Ge et al., 2018)
	ICR mice 1. Female mice (8 weeks old and ovariectomized) 2. Male mice (10 weeks old and orchidectomized)	Six weeks after the mice were ovariectomized or orchidectomized, the mice were randomly divided into 6 groups with 12 mice each. The mice were treated intragastrically once a day for 8 weeks before being sacrificed. 1. Control group: sham-operated mice 2. Model group: ovariectomized/orchidectomized mice 3. Female positive drug group: females treated with estradiol valerate (E2) 4. Male positive drug group: males with alendronate sodium (AS) 5. Psoralen group: mice treated with psoralen for both gender groups 6. Angelicin group: mice treated with angelicin for both gender groups	1. Psoralen group: 10 and 20 mg/kg 2. Angelicin group: 10 and 20 mg/kg	1. Female group: (a) No changes in the ALP in the serum were seen even in between the control and model groups. (b) Significant decrease in TRACP in the serum was seen in mice treated with 20mg/kg angelicin. However, there were no changes seen between control and model group. (c) Significant increase in the ALP/TRACP ratio in the serum were seen in the mice treated with E2, 10 mg/kg psoralen and 10mg/kg angelicin. However, there were no changes seen between control and model group. (d) Significant increase of CTX-1(degraded type I collagen) in the serum model group as compared to the control group. However, the effect was significantly attenuated by E2, 20 mg/kg psoralen, 10 mg/kg angelicin and 20 mg/kg angelicin. (e) No changes in OCN in the serum were seen even in between the control and model groups. (f) Significant increase in the degree of anisotrophy (DA) was seen in the model group compared to the control group but the effect was reversed by E2, 10 mg/kg psoralen, 10mg/kg angelicin and 20mg/kg angelicin. (g) Significant decrease in bone volume (BV/TV) in model group compared to the control group but were attenuated by E2 and 20 mg/kg psoralen. (h) Significant decrease in trabecular number (Tb.N) in model group compared to the control group but were attenuated by all treatments. (g) Significant increase in trabecular thickness (Tb.Th) in model group compared to the control group but were attenuated by all treatments. (h) Significant increase in trabecular separation (Tb.Sp) in model group compared to the control group but were attenuated by all treatments. (i) Significant decrease in bone strength in model group compared to the control group but was attenuated by all treatments. 2. Male group: (a) Significant increase in ALP in male mice was seen when treated with 10mg/kg psoralen and angelicin. However, there were no changes seen between the control and model groups. (b) No changes were seen in the TRACP in the serum even in between the control and model groups. (c) Significant increase in the ALP/TRACP ratio in the serum was seen in the mice treated with 10 mg/kg psoralen and 10 mg/kg angelicin. However, there were no changes seen between control and model group. (d) Significant increase in CTX-1 in the serum in the model group. However, the effect was significantly by AS, 10 mg/kg psoralen, 10 mg/kg angelicin and 20 mg/kg angelicin. (e) Significant increase in the OCN in the serum model group as compared to the control group. However, the effect was significantly attenuated by 10 mg/kg angelicin. (f) Significant increase in the DA were seen in the model group compared to the control group but the effect was reversed by 10 and 20 mg/kg psoralen and angelicin. (g) Significant decrease in bone volume (BV/TV) in model group compared to the control group but were attenuated by all treatments. (h) Significant decrease in trabecular number (Tb.N) in model group compared to the control group but were attenuated by all treatments. (g) Significant increase in trabecular thickness (Tb.Th) in model group compared to the control group but were attenuated by 20 mg/kg psoralen. (h) Significant increase in trabecular separation (Tb.Sp) in model group compared to the control group but were attenuated by all treatments. (i) Significant decrease in bone strength in model group compared to the control group but was attenuated by all treatments.	(Yuan et al., 2016)
	6 week old female Sprague Dawley rats (weight: 140–160g)	The rats were distributed into 4 groups before ovariectomizing. The rats were treated with angelicin every 3 days for 12 weeks. 1. Control group: sham-operated 2. Model group: ovariectomized mice (osteoporosis) 3. 10 mg/kg angelicin group 4. 20 mg/kg angelicin group	10 and 20 mg/kg angelicin	1. Significant decrease in calcium/creatine (Ca/Cr) levels in urine: ≥ 10 mg/kg angelicin 2. Significant increase in bone mineral density (BMD): ≥ 10 mg/kg angelicin 3. Significant decrease in structure score of the proximal tibial metaphysis (PTM): ≥ 10 mg/kg angelicin 4. Significant increase in serum leptin levels: ≥ 10 mg/kg angelicin 5. Significant decrease in serum calcium levels: 20 mg/kg angelicin 6. Significant decrease in ALP activity in blood: ≥ 10 mg/kg angelicin 7. Significant decrease in COL I, OCN and OPN mRNA in total cartilage tissue: ≥ 10 mg/kg angelicin 8. Significant decrease in MDA activity in blood: ≥ 10 mg/kg angelicin 9. Significant increase in SOD, GSH and GSH-PX activity in blood: ≥ 10 mg/kg angelicin 10. Significant decrease in caspase 3 and 9 activity: ≥ 10 mg/kg angelicin 11. Significant increase in WNT protein expression: ≥ 10 mg/kg angelicin 12. Significant increase in β-catenin protein expression: ≥ 10 mg/kg angelicin 13. Significant decrease in PPARγ protein expression: ≥ 10 mg/kg angelicin	(Wang et al., 2018)
Anti-periondontitis	1. *Porphyromonas gingivalis* (*P. gingivalis*) 2. THP-1 cells 3. Primary human periodontal ligament cells (hPDLCs) obtained from patients using tissue explant method. 4. *In vivo*: Wild type 8 week old male C57BL/6 mice	*In vitro* assays: (a) Incubation for 48 h under anaerobic conditions for MBC, MIC, biofilm formation assays (b) Incubation for 24 h in assays to assess biofilm reduction, viability, and thickness (c) Incubation for 24 h in MTT assay for hPDLCs and THP-1 cells (d) Pretreatment for 2 h on THP-1 cells before inflammatory stimulation with *P. gingivalis*-derived lipopolysaccharide (Pg-LPS) (e) Treatment for 9 days on hPDLCs in osteogenic induction assay. On the 3rd day, the expression of osteogenic proteins was analyzed *via* measurement of concentration in BCA protein assay kit. *In vivo* assays: (a) Mice were injected with angelicin 30 min prior to injection with Pg-LPS. The mice were injected with angelicin and 10 mg/ml Pg-LPS three times a week for 4 weeks.	1. *In vitro* assays: (a) 1.5625, 3.125, 6.25, 12.5, 25, and 50 µg/ml angelicin and psoralen in bacterial, cell viability (on THP-1 and hPDLCs) and inflammatory analysis (b) 6.25 µg/ml of psoralen and 3.125 µg/ml angelicin in osteogenic induction analysis 2. *In vivo* assays: 20mg/ml angelicin	1. *In vitro* assays: (a) Minimum inhibitory concentration (MIC) (lowest concentration that shows no macroscopically visible bacterial growth at 625 nm) of angelicin: 3.125 μg/ml •MIC of psoralen: 6.25 μg/ml (b) Minimum bactericidal concentration (MBC) (lowest concentration where no bacterial clone grew on the agar) of angelicin: 50 μg/ml •MBC of psoralen: 50 μg/ml (c) Minimum biofilm inhibition concentration (MBIC_50_) (lowest drug concentration that results in 50% inhibition of formation of biofilm compared to control) of angelicin: 7.5 μg/ml • MBIC_50_ of psoralen: 15.8 μg/ml (d) Minimum biofilm reduction concentration (MBRC^50^) (lowest drug concentration that reduces biofilm to 50% compared to control) of angelicin: 23.7 μg/ml • MBRC_50_ of psoralen: 24.5 μg/ml (e) Sesemile MIC (SMIC_50_) (lowest concentration causing 50% reduction in bacterial viability compared to untreated control) of angelicin: 6.5 μg/ml • SMIC_50_ of psoralen: 5.8 μg/ml (f) Angelicin has shown more significant decrease in thickness of biofilm (p < 0.001) as compared to psoralen (p < 0.01) (g) Significant decrease in THP-1 and hPDLCs cell viability for both psoralen and angelicin: ≥ 2.5 μg/ml (h) Significant decrease of IL-1β mRNA expression by angelicin: 1.5625 μg/ml (p < 0.001) • Significant decrease of IL-1β mRNA expression by psoralen: 1.5625 μg/ml (p < 0.05) (i) Significant decrease in IL-8 mRNA expression by angelicin: 1.5625 μg/ml (p < 0.001) • Significant decrease in IL-8 mRNA expression by psoralen: 3.125 μg/ml (p < 0.01) (j)Significant decrease in IL-1β protein expression by angelicin: 1.5625 μg/ml (p < 0.05) • Significant decrease in IL-1β protein expression by psoralen: 3.125 μg/ml (p < 0.05) (k) Significant decrease in IL-8 protein expression by angelicin: 3.125 μg/ml (p < 0.05) • Significant decrease in IL-8 protein expression by psoralen: 6.25 μg/ml (p < 0.05) (l) Significant increase in RUNX2, DLX5, and OPN mRNA expression hPDLCs as compared to control by both angelicin and psoralen: Day 3, 6, and 9 2. *In vivo* assays: (a)Significant in bone volume percentage (bone volume/total volume, BV/TV), bone mineral density (BMD) and bone surface/bone volume (BS/BV) by angelicin	(Li et al., 2018)
Pro-chondrogenesis	Pre-chondrogenic ATDC5 cells	1. Measurement of the rate of cell growth with MTT assay: 24 h incubation with angelicin 2. Induction of chondrogenic differentiation: Incubation of cells with angelicin for 14 days 3. Measurement of chondrogenic marker genes after treatment with angelicin for 7, 14 and 21 days 4. Measurement of ALP activity of cells treated with angelicin for 7, 14 and 21 days 5. Measurement of BMP-2 protein level after treatment with angelicin for 1, 2 and 3 days. 6. Measurement of JNK, ERK and p38 protein levels (including phosphorylated proteins) after cells were serum-starved for 16 h and then treated with angelicin for 1.5, 3, 6, 12, and 24 h.	1. Rate of cell growth with MTT assay: 0.001, 0.005, 0.05, 0.1, and 1 μM angelicin 2. Induction of chondrogenic differentiation (formation of cartilage nodules): 0.01, 0.05, 0.5, and 1μM angelicin 3. Measurement of collagen X and collagen II mRNA for 21 days: 0.005, 0.05, 0.5, and 1 µM 4. Measurement of BSP, collagen I, RUNX2, collagen II, collagen X, OCN, β-catenin, smad 4, and SOX9 mRNA for 21 days: 0.05 µM 5. Analysis of the ALP activity in cells: 0.05 µM angelicin 6. Measurement of BMP-2 protein level: 0.05 µM angelicin 7. Measurement of MAPK kinase protein levels: 0.05 µM angelicin	1. No increase in the rate of cell growth was observed after treatment with angelicin. 2. Significant increase in number of stained cartilage nodules (synthesis of matrix proteoglycan): 0.01, 0.05, and 0.5μM angelicin 3. Increase in collagen X and collagen II mRNA: ≥0.005 μM angelicin 4. Increase in BSO, collagen I and RUNX2 mRNA: day 14 5. Increase in collagen II, collagen X, OCN, β-catenin, smad 4, and SOX9 mRNA: ≥ day 7 6. Increase in ALP activity in cells treated with angelicin: ≥ day 14 7. Increase in BMP-2 protein expression after 24 h 8. Increase in phosphorylated ERK and p38 at 1.5 h after treatment with angelicin.	(Li et al., 2012)
Anti-viral	1. African green monkey kidney (CV-1) cells infected with HSV 2. Human normal skin (KD) cell line (CRL 1295) infected with HSV 2. Xeroderma pigmentosum (XP) cell line (CRL 1223) infected with HSV	Pretreatment of angelicin or 8-MOP for 30 min before being irradiated with 1.6 KJm^-2^min^-1^ at 365nm UVA	1. 50 µM angelicin or 8-MOP for CV-1 cells 2. For human skin cells, 0.3mM angelicin and 50µM 8-MOP	1. 8-MOP is 7.5 fold higher than angelicin in reducing the HSV production capacity in CV-1 cells with UVA. 2. For the first 12 h of treatment of angelicin with 365nm UVA, the viral yield decreases and then starts to recover after 24 h and finally reaches its maximum yield after 72 h in CV-1 cells. Similar results were seen with 8-MOP with the exception that an initial decrease and then an increase in HSV production, reaching a maximum at 72 h was seen. 3. In normal and XP cell lines, angelicin is 5.4 and 4.1 less efficient than 8-MOP, respectively. The HSV production is more inhibited in XP cells as compared to normal cells when treated with angelicin. 4. The amount of unscheduled DNA synthesis induced by 8-MOP with UVA is 40% higher than angelicin with light.	(Coppey et al., 1979)
	1. Mouse 3T3 cell line infected with murine cytomegalovirus (MCMV)	Virus suspensions were incubated with the compounds for 30 min before exposing UVA irradiation (incident energy = 300 W/m^2^) for 30 min.	10 µg/ml of angelicin	1. Angelicin did not produce cross-links in the viral DNA but its phototoxicity suggests that monoadducts may cause viral genome to be non-infectious.	(Altamirano‐Dimas et al., 1986)
	1. Mouse 3T3 cell line infected with murine cytomegalovirus (MCMV) and sindbis virus (SV) 2. *Eschericia coli* infected with bacterial phage T4 and phage M13	Virus suspensions were incubated with the compounds for 30 min before UVA irradiation (incident energy = 5 W/m^2^).	(Not mentioned)	Angelicin has relative toxicity against double stranded DNA phage T4, double stranded DNA MCMV, single stranded DNA phage M13, and single stranded RNA SV.	(Hudson and Towers, 1988)
	1. BHK21 cells (baby hamster kidney fibroblast) containing MHV-68 virus 2.Vero cells (green monkey kidney) containing MHV-68 virus 3. BC-3 cells (KSHV positive, EBV negative cells) 4. BCBL-1 cells (KSHV positive, EBV negative cells) 5. B95.8 cells (EBV positive, KSHV negative) 6. Raji cells (EBV positive, KSHV negative) 7. BC-3G cells (containing strong RTA-responsive element which drives destabilized EGFP expression) 9. 293T cells for promoter reporter analysis	1. Antiviral screening: Pretreatment of compounds 3 h prior to viral infection and during 1 h of viral adoption in BHK21 cells 2. Analysis of promoter reporter: 294T cells were transfected with 650 ng of plasmid and then angelicin was used to treat 1 h after post-transfection. 3. Plaque reduction assays: compounds were added as a pretreatment 3 h prior to viral adoption or as a post-treatment in the media. 4. Cytotoxic assay: angelicin was incubated with cells for 24 h before MTT assay.	1. Anti-viral screening for compounds: 4, 20 and 100µg/ml of compounds against MHV-68 2. To reconfirm the anti-viral effect of angelicin on viral replication= 3,7.5, 15, and 30 µg/ml before and after viral infection.) 3. To determine antiviral efficacy: 0.1–90 µg/ml (0.54–483.3µM) angelicin 4. To determine cytotoxicity toward Vero cells: 0.1 to 500 µg/ml (0.54 to 2,685.86 µM) •Positive control: GCV 20 µg/ml	1. Angelicin reduces MHV-68 viral replication dose-dependently. 2. Angelicin inhibits ORF45 and ORF65 late gene protein expressions dose-dependently. • GCV inhibits completely the expression of both proteins. 3. Angelicin decreases viral genome replication dose-dependently. 4. Angelicin directly inhibits mRNA expression of RTA dose-dependently by reducing the transactivation of RTA promoter. 5. Angelicin reduces plaque formation of MHV-68 by about 80% as compared to negative control post-treatment. • GCV reduces plaque formation by 100% in pre and post-treatments. 6. Concentration of angelicin required to inhibit MHV-68 replication by 50% (IC50): 5.39 µg/ml (28.95 µM) 7. Concentration of angelicin to decrease cell viability to 50% (CC50): Not obtainable. At 500 µg/ml (2,685.86 µM) angelicin, cell viability is at 72.5% 8. Angelicin efficiently inhibits EBV lytic replication *via* inhibition of early antigen diffuse (EA-D) expression, relative EBV viral genome loads and expression of BMRF1 (EBV early lytic transcript gene) mRNA levels in Raji and B95.8 cells. 9 Angelicin significantly reduces the number of KSHV lytic replication *via* inhibition of RTA protein expression and reducing KSHV viral genome loads in BC-3G and BCBL-1 cells. 10. Angelicin is more effective in reducing MHV-68 plaque formation as compared to psoralen. 11. Psoralen is more effective in reducing KSHV lytic replication as compared to angelicin. 12. Angelicin and psoralen are equal in reducing EBV lytic replication.	(Cho et al., 2013)
Erythroid differentiation	1. Human leukemic K562 cell line 2. two-phase liquid culture of human erythroid progenitors isolated from peripheral blood samples of normal donors	1. Angelicin was incubated with K562 cells for up to 7 days to determine the effect of angelicin on the proliferation and differentiation of the cells. 2. K562 cells were incubated with angelicin for 5 days to determine the mRNA content of γ and α- globin. 3. Compounds were added on day 4–5 of phase II culture in two-phase liquid culture before harvesting the cells on day 12.	1. K562 cell proliferation: 0.05, 0.1, 0.2, 0.4, 0.8, and 1mM angelicin 2. K562 cell differentiation: 50, 100, 200, and 400μM angelicin 3. Determination of γ and α-globin mRNA expression level in K562 and two-phase culture: 200μM angelicin • 120 μM hydroxyurea was used as a positive control in Determination of γ and α-globin mRNA expression level in two-phase culture	1. Angelicin caused dose-dependent inhibition of cell proliferation and differentiation of K562 cells 2. Differentiation of K562 cells based on the percentage of benzidine positive cells: (a) Angelicin at 400 μM = 60.6 ± 6.2 (b) Angelicin at 200 μM = 48.7 ± 7.7 (c) Cytosine arabinoside (Ara-C) at 1 μM = 78.3 ± 4.1 (d) Mithramycin at 50 mM = 85.6 ± 7.2 (e) Cisplatin at 6 μM = 62.5 ± 7.8 (f) Butyric acid at 2.4 mM = 35.3 ± 3.7 3. Angelicin induces 44.9 fold increase as compared to ara-C (18-fold increase) in γ-globin mRNA in K562 cells 4. Angelicin induces high increase of γ-globin mRNA and a low increase in α-globin mRNA. The increase in γ-globin for angelicin is 20.4 fold induction as compared to hydroxyurea that only increased induction by 6.6 fold. 5. HPLC analysis of HbF in angelicin treated cultures increased from 1.4 ± 0.6% in control cells to 11.2 ± 3.8%. Hydroxyurea increased to 4.8 ± 0.9%.	(Lampronti et al., 2003)
	1. Human leukemic K562 cells 2. two-phase liquid culture isolated from peripheral blood samples of normal donors	1. Pre-irradiated solutions of 5 -MOP, 8-MOP, and angelicin with 16 Jcm-2 UVA were incubated with K562 cells for 5, 6, and 7 days. 2. Pre-irradiated solutions of 5-MOP, 8-MOP, and angelicin with UVA with 16 Jcm-2 were added on day 4-5 of phase II and cells were harvested on day 7.	5, 10, 15, and 20 μM of compounds	1. Angelicin photoproducts increase erythroid differentiation dose-dependently. A modest reduction of cell viability is seen dose-dependently after incubation for 6 days. 2. The erythroid differentiating properties of angelicin photoproducts is significantly reduced under anaerobic conditions compared to aerobic conditions. 3. Angelicin photoproducts significantly increase HbA and HbF after 7 days of incubation in two-phase culture system. HbF fold increase is higher than HbA fold increase. Angelicin photoproducts induced a higher fold increase in both HbA and HbF as compared to 5-MOP and 8-MOP.	(Viola et al., 2008)

## Natural Occurrence of Angelicin

Both psoralen and angelicin can be found in plants from the Leguminosae (Fabaceae), Apiaceae (Umbelliferae) and Moracea family. However, not all plants produce both these furocoumarins. In the Rutaceae family, which encompasses the citrus fruits, only linear isomers were produced (Pathak et al., 1962; Zobel and Brown, 1991). No plant has so far been found that produces only angular furocoumarin; the plants studied so far have always found to be either produce both isomers or linear isomers alone. This finding suggests that angular furocoumarins came later from an evolutionary viewpoint than linear furocoumarins (Dueholm et al., 2015). The existence of angular furocoumarin was hypothesized to be an evolutionary advantage over insects that can metabolize linear furocoumarins. An example is the larvae of *Papilo polyxenes* or the Black Swallowtail butterfly that produces microsomal cytochrome P450 monooxygenases (P450s)- an enzyme that can catalyze the metabolism of linear furocoumarins. However, angular furocomarins such as angelicin can bind to the active sites of these enzymes, inhibiting the enzyme from metabolizing the toxic linear furocoumarin (Ma et al., 1994; Wen et al., 2006). This shows that there was an evolutionary need for plants to develop angular furocoumarin, even though it is less cytotoxic than its sister isomer. In this review, the list of plants that produce angelicin is tabulated in **Table 2**. Other extracted compounds are also included. From the table, it can be seen that the percentage yield of angelicin and the other compounds not only changes with the type of plants but also with the part of plant and season, suggesting as mentioned above that these compounds could be produced to act as a defence system against diseases or pests. Other than that, the other listed plants were also used traditionally as medicine. However, whether the compounds contribute individually or accumulatively to the traditionally believed “medicinal” attributes of the plant is still unclear and under research.

**Table 2:** Plants containing angelicin and its traditional uses.

Plant	Family	Geographical location	Parts containing angelicin	Percentage of reported compounds	Traditional medicine uses	References
***Angelica shikokiana***	*Umbelliferae/Apiaceae*	Japan	Aerial parts	1. α-glutinol (0.0097% 68mg) 2. β-amyrin (0.0014%, 10mg) 3. Isoxypteryxin (0.0017%, 12mg) 4. Isoepoxypteryxin 0.26%, 1.8g) 5. Angelicin (0.0043%, 30mg) 6. β-sitosterol glucoside (0.005%, 35mg) 7. Bergapten (0.0014%, 10mg) 8. Psoralen (0.0019%, 13mg) 9. Hyuganin C (0.001%, 7mg) 10. Hyganin E (0.002%, 14mg) 11. Hydroxymethyl-2-furaldehyde (0.001%, 7mg) 12. Kaempferol (0.0086%, 60mg) 13. Luteolin (0.0073%, 51mg) 14. Methyl chlorogenate (0.005%, 35mg) 15. Chlorogenic acid (0.0023%, 16mg) 16. Quercetin (0.0041%, 29mg) 17. Kaempferol glucoside (0.0087%, 61mg) 18. Kaempferol rutinoside (0.0014%, 10mg) 19. Adenosine (0.0014%, 10mg) *Percentage calculated based on 10kg powdered dried aerial parts	Treating digestive and circulatory systems diseases	(Mira and Shimizu, 2015; Mira and Shimizaru, 2016)
***Angelica sylvestris* L. var. *sylvestris***	*Umbelliferae/Apiaceae*	Europe and Asia Minor	Aerial parts	1. Angelicin (0.31 ± 0.015 mg/100g) 2. Imperatorin (2.36 ± 0.033 mg/100g) *Compounds were extracted from aerial parts of the plant.	Stimulate appetite, treat anorexia, anaemia, vertigo, influenza, dizziness, migraine, and bronchitis, relieve cough, sore throat, indigestion and cold	(Sarker et al., 2003; Mira and Shimizu, 2016; OrHan et al., 2016)
***Bituminaria basaltica***	*Fabaceae/Leguminosae*	Italy	Aerial parts	1. angelicin (0.0008%, 10mg) 2. psoralen (0.0002%, 3mg) 3. plicatin B (0.0005%, 6mg) 4. erybraedin C (0.0037%, 46mg) 5. 3,9-dihydroxy-4-isoprenyl-pterocarpan (0.0014%, 17mg) 6. isoorientin (0.0003%, 4mg) 7. daidzin (0.0005%, 6mg) 8. bitucarpin A (0.001%, 12mg) *Percentage calculated based on 1220g air dried and powdered aerial parts	Disinfection to treat wounds, hair restoration, urinary infections	(Minissale et al., 2013; Bandeira Reidel et al., 2017)
***Bituminaria bituminosa. L***	*Fabaceae/Leguminosae*	Mediteranian Europe and Maaronesia	Leaves and stems	Average mean concentration of compounds in dry matter from 7 populations: (a) Leaves: 1. Angelicin (4.59 g/kg in the summer; 6.58g/kg in the autumn) 2. Psoralen (6.15g/kg in the summer; 5.59g/kg in the autumn) 3. (E)- werneria chromena (0.15g/kg in the summer; 0.10g/kg in the autumn) 4. Plicatin B (2.10g/kg in the summer; 0.99g/kg in the autumn) 5. Bitucarpin A (1.32g/kg in the summer; 1.49g/kg in the autumn) 6. Morisianin (0.02g/kg in the summer; 0.01g/kg in the autumn) 7. Erybraedin C (0.57g/kg in the summer; 0.27g/kg in the autumn) (b) Stems: 1. Angelicin (2.37g/kg in the summer) 2. Psoralen (2.55g/kg in the summer)	Vulnerary, disinfectant and cicatrising	(Azzouzi et al., 2014; Pecetti et al., 2016)
***Bituminaria morisiana***	*Fabaceae/Leguminosae*	Sardinia Italy	Seed	1. Morisianine (0.003%, 2.7mg) 2. Erybraedin C (0.018%, 18.5mg) 3. Angelicin (0.014%, 13.8mg)4. Psoralen (0.006%, 5.6mg) *Percentage calculated based on 100.9g air-dried and powdered seeds	(Not found)	(Pistelli et al., 2003; Leonti et al., 2010)
***Cicuta virosa* Linnaeus** **(water hemlock)**	*Umbelliferae/Apiaceae*	Russia, Japan, China	Dried whole plant	1. 11,110 -dimer of scopoletin (0.83%, 25mg) 2. 11-O-b-glucopyranosylhamaudol (1.17%, 35.2mg) 3. Isobyakangelicin (1.54%, 46.3mg) 4. Psoralene (1.27%, 38.1mg) 5. Angelicin (2.02%, 60.5mg) 6. Prim-O-glucosylangelicain (1.44%, 43.1mg) 7. Apiosylskimmin (1.86%, 55.8mg) 8. Rutin (1.18%, 35.3mg) 9. Quercetin-3-Ob-D-rhamnoside (2.61%, 78.3mg) *Percentage calculated based on 3kg air dried powder plant material	Lethal poisoning toward humans	(Uwai et al., 2000; Tian et al., 2011; Wang et al., 2011; Wang et al., 2016)
***Ficus carica*** **(Common Fig)**	*Moracea*	Tropical and sub-tropical countries	Fruit	(A) Volatile compounds extracted from figs of *Ficus carica* by Gibernau and team. Compounds labelled with an asterisk are those that were tentatively identified with mass spectrophotometer library. The yield and original weight of the compounds were not reported. 1. Benzyl aldehyde (2 isomers) 2. Benzyle alcohol 3. Furanoid (cis) linalool oxide 4. Furanoid (trans) 5. Pyranoid (cis) linalool oxide 6. Pyranoid (trans) 7. Cinnamic aldehyde 8. Indole 9. Cinnamic alcohol 10. Eugenol 11. trans-Caryophyllene 12. Sesquiterpene 1 13. Sesquiterpene 2 14. Sesquiterpene 3 15. Sesquiterpene 4 16. Sesquiterpene 5 17. Hydroxycaryophyllene 18. Oxygenated sesquiterpene 1 19. Oxygenated sesquiterpene 2 20. Angelicin* 21. Bergapten* (B) Coumaric compounds obtained in the month of June only and their relative concentrations based on FID peak areas of n-hexane or dichloromethane fraction by Marrelli and team. 1. Psoralen (23.30%) 2. 8-methoxypsoralen (3.65%) 3. Angelicin (2.5%) 4. Bergapten (15.20%) 5. Rutaretin (21.10%) 6. Pimpinellin (1.9%) 7. Seselin (19.5%) *Weight of *Ficus* samples for extraction: 300g *List of relative concentration (based on FID peak fractions) for major fatty acids, sterols and terpenes obtained from the month of June, July and September can be viewed in the original article by Marrelli and team.	Anti-pyretic, aphrodisiac, inflammation, paralysis, purgative, control haemorrhages	(Gibernau et al., 1997; Marrelli et al., 2012)
***Heracleum maximum***	*Umbelliferae/Apiaceae*	North America	Roots	1. (3R,8S)-Falcarindiol (0.037%, 37mg)2. Bergapten (0.015%, 15mg)3. Isobergapten (0.031%, 31mg)4. Angelicin (0.005%, 5mg)5. Sphondin (0.023%, 23mg)6. Pimpinellin (0.029%, 29mg)7. Isopimpinellin (0.033%, 33mg)8. 6-Isopentenyloxyisobergapten (%, 1mg) *Percentage calculated based on 100g freeze dried roots	Infectious diseases and respiratory ailment including tuberculosis	(O׳Neill et al., 2013; Bahadori et al., 2016)
***Heracleum meoellendorffi***	*Umbelliferae/Apiaceae*	Korea and China	Roots	1. Angelicin (21.43%, 300mg) 2. Isobergapten (12.14%, 170mg) 3. Pimpinellin (41.43%, 580mg) 4. (3S, 4R)-3, 4-epoxypimpinellin (14.07%, 197mg) *Percentage calculated based on 1.4kg air dried roots	Common cold, headache and analgesics	(Bahadori et al., 2016; Yeol Park et al., 2017)
***Heracleum persicum*** **(Persian hogweed)**	*Umbelliferae/Apiaceae*	Iran	Roots	1. Psoralen (0.013%, 2mg) 2. Bergapten (0.106%, 15.9mg) 3. Xanthotoxin (0.117%, 17.6mg) 4. Isopimpinellin (0.089%, 13.3mg) 5. Angelicin (0.017%, 2.5mg) 6. Isobergapten (0.135%, 20.3mg) 7. Sphondin (0.085%, 12.8mg) 8. Pimpinellin (0.371%, 55.6mg) 9. Beratomin (0.039%, 5.8mg) 10. 5-methoxyheratomin (0.015%, 2.3mg) 11. Moellendorffiline (0.038%, 5.7mg) 12. Fraxetin (0.011%, 1.7mg) *Percentage calculated from 15g n-hexane extract of dried and grounded roots	Anti-flatulence, digestive, anti-infection, pain killer and tonic agent	(Bahadori et al., 2016; Dehghan et al., 2017)
***Heracleum platytaenium***	*Umbelliferae/Apiaceae*	Turkey	Aerial parts	1. Xanthotoxin (2.97 ± 0.019 mg/100g) 2. Angelicin (1.74 ± 0.033 mg/100g) 3. Isopimpinellin (0.31 ± 0.003 mg/100g) 4. Bergapten (2.51 ± 0.045 mg/100g) 5. Pimpinellin (7.73 ± 0.159 mg/100g) 6. Osthol *Compounds were extracted from aerial parts of the plant.	Gastric, epilepsy, enteritis	(Dincel et al., 2013; Bahadori et al., 2016; OrHan et al., 2016)
***Heracleum rawianum***	*Umbelliferae/Apiaceae*	Iran	Aerial parts of the plant	1. Angelicin (0.12%, 5.9g) 2. Allobergapten (0.00056%, 28mg) 3. Sphondin (0.00066%, 33mg) *Percentage calculated based on 5kg aerial parts of the plant	Antiseptic, carminative, digestive and analgesic	(Mahmoodi Kordi et al., 2015; Bahadori et al., 2016)
***Heraculeum thomsoni***	*Umbelliferae/Apiaceae*	India	Aerial parts	1.Angelicin (0.0138% yield) 2. Psoralen (not mentioned) 3. heratomin (not mentioned) 4. Sphondin (not mentioned) 5. Bergaptol (not mentioned) 6. Apterin (not mentioned) *Weight of the aerial parts of the plant: 2.05kg	(Not found)	(Patnaik et al., 1988)
***Pastinaca sativa*** **(parsnip)**	*Umbelliferae/Apiaceae*	North America	Leaf, seeds, roots, and shoots	(A) Biosynthethic pathway of angelicin and psoralen were studied in the leaves and seeds by Munakata and team. (B) Compounds extracted from the roots and shoots. The yield or initial plant weight was not mentioned by Lohman and McConnaughay. 1. Angelicin 2. Bergapten 3. Imperatorin 4. Isopimpinellin 5. Xanthotoxin 6. Sphondin	Sudorifics and diuretics	(Baskin and Baskin, 1979; Hendrix, 1984; Berenbaum et al., 1991; Lohman and McConnaughay, 1998; Waksmundzka-Hajnos et al., 2004; Munakata et al., 2016)
***Pleurospermum brunonis***	*Umbelliferae/Apiaceae*	Pakistan	Aerial parts	1. 5-O-β-D-glucopyranosyl-6-methoxyangelicin (0.001%, 21mg) 2. Ferulic acid (0.0015%, 29mg) 3. Angelicin (0.0045%, 89mg) *Percentage calculated from 2kg air dried and powdered aerial parts of the plant	Fever, analgesic, headache	(Sharma et al., 2005; Ikram et al., 2015; Rather et al., 2017)
**Psoralea corylifolia L**.	*Fabaceae/Leguminosae*	Malay peninsula, Indonesia, China, Taiwan	Fruit	(A) Yield reported by Lin and team 1. Bakuchiol (0.85%, 8.5g) 2. Psoralen (0.32%, 3.2g) 3. Angelicin (0.34%, 3.4g) *Percentage calculated based on 1kg pulverized plant (part of the plant was not mentioned) were (B) Yield reported by Chen and team 1. 7-O-Methylcorulifol A (0.00012%, 4.5mg) 2. 7-O-Isoprenylcorylifol A (0.00012%, 4.5mg) 3. 7-O-Isoprenylneobavaisoflavone (0.00011%, 4.1mg) 4. Angelicin (0.0027%, 10.4mg) 5. Psoralen (0.0001%, 3.9mg) 6. Bakuchiol (0.00011%, 4.3mg) 7. 12,13-dihyro-12,2-epoxybakuchiol (0.00012%, 4.5mg) 8. p-hydroxybenzaldehyde (0.0002%, 7.6mg) 9. Bavachalcone (0.000095%, 3.6mg) 10. Psoralidin (0.00012%, 4.5mg) 11. β-sitosterol 12. Stigmasterol (Combination β-sitosterol and stigmasterol: 0.0035%, 128mg) *Percentage calculated based on 3.8kg dried fruit	Spermatorrhea, backache, vitiligo, callus, knee pain, pollakiuria, enuresis, premature ejaculation, alopecia, psoriasis, asthma, nephritis	(Prasad et al., 2004; Lin et al., 2007; Choi et al., 2016; Chen et al., 2017)
***Sanicula lamelligera Hance*** **(Fei-Jing-Cao)**	*Umbelliferae/Apiaceae*	North America and China	Dried whole plant	1. Angelicin (0.00016%, 40mg) 2. Isoferulaldehyde (0.00002%, 5.1mg) 3. Hydroxybakuchiol (0.00011%, 27mg) 4. 22-angeloyl-R_1_-barrigenol (0.000013%, 3.2mg) 5. 5, 6, 7, 8, 4'-pentamethoxyflavone (0.00017%, 42.6mg) 6. 3, 5, 6, 7, 8, 3',4'-heptamethoxyflavone (0.000053%, 13.2mg) 7. Isobavachin (0.00012%, 30.5mg) 8. Isoliquiritigenin (0.000018%, 4.5mg) 9. Furano(2”, 3”, 7, 6)-4'-hydrpxyflavone (0.000036%, 9mg) *Percentage based on dried whole plant 25kg	Colds, asthma, bleeding, cough, gall, chromic chest pains, mild cattarhs, wounds, and amenorrhea	(Naylor et al., 2011; Zhou et al., 2014; Xu et al., 2015)

**Figure 4:**
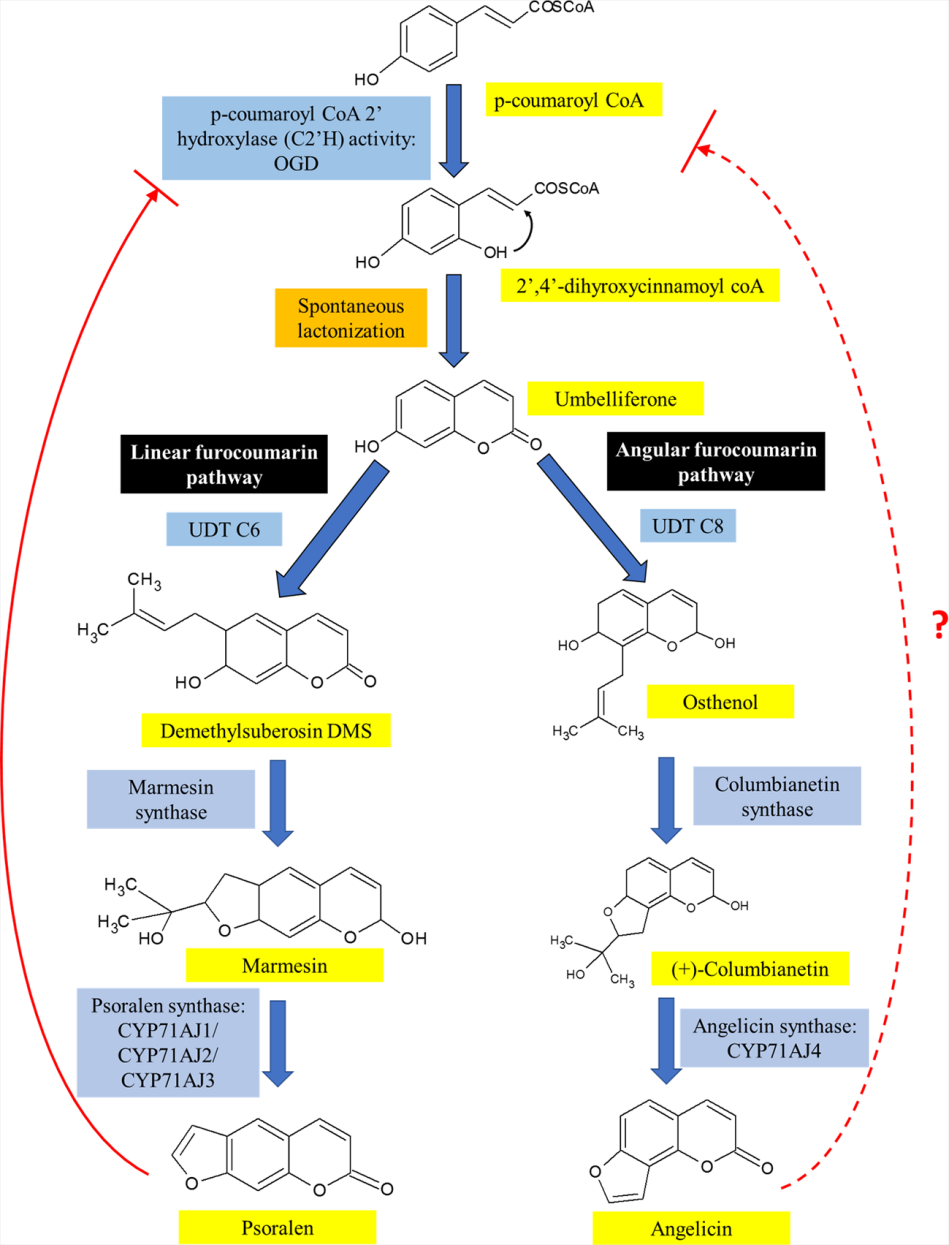
The biosynthesis pathway of linear and angular furocoumarin in plants. Both psoralen and angelicin come from the same precursor, umbelliferone, which had been modified differently to form both linear and angular furocoumarin pathways. Though psoralen is able to act as a negative feedback against C2'H activity, it is not known yet if angelicin is also involved in the negative feedback loop.

A summary of the biosynthesis of both angelicin and psoralen is illustrated in **Figure 4**. Umbelliferone is the precursor compound of both linear and angular furocoumarins. Umbelliferone is an intermediate 7-hydroxycoumarin derived from p-coumaryl CoA that has undergone ortho-hydroxylation *via* p-coumaroyl coA2'hyroxylase (C2'H) activity. In *Ruta graveolens L*. 2-oxoglutarate-dependent dioxygenase (OGD) was found to synthesise 2,4-dihydroxycinnamoyl CoA which then transforms into umbelliferone after spontaneous closure of the lactone ring under acidic or neutral conditions independent of enzymes. Further investigation had also discovered that psoralen acts as negative feedback against C2'H activity to prevent excessive production of psoralen in the plant (Vialart et al., 2012). It is not known if angelicin is also involved in the negative feedback loop against C2'H activity.

After umbelliferone, the distinction of both linear and angular furocoumarin biosynthesis happens based on the prenylation position of umbelliferone at C6 or C8 by umbelliferone dimethylallyltransferase (UDT). UDT is a prenyltransferase that synthesizes demethylsuberosin (DMS) at C6 position of umbelliferone and ostenol at the C8 position (Karamat et al., 2013; Munakata et al., 2016). The synthesis of DMS would lead to the formation of psoralen while osthenol is the precursor of angelicin. DMS is then catalyzed by both marmesin synthase and psoralen synthase to form psoralen itself (Hamerski and Matern, 1988; Larbat et al., 2008; Roselli et al., 2017). On the other hand, for angular furocoumarins, osthenol is catalyzed by columbianetin synthase to form (+)-columbianetin (Roselli et al., 2017). Angelicin synthase then catalyzes the conversion of (+)-columbianetin to angelicin through the abstraction of syn-C3'-hydrogen (Larbat et al., 2008). The first to discover angelicin synthase were Larbat and colleagues who isolated the parsnip variant of angelicin synthase (CYP71AJ4) together with its complementary psoralen synthase, CYP71AJ3, using the genomic sequence of psoralen synthase isolated from *Ammi majus* (Larbat et al., 2008; Munakata et al., 2016). They also discovered that the genes for angelicin synthase and psoralen synthase from the CYP71AJ subfamily of parsnip share 70% similarity with each other, but the portions were believed to code for active substrate sites sides only showed 40% similarity (Larbat et al., 2008). A recent journal article reported that using bacterial artificial chromosome (BAC) library, it was identified that both *CYP71A3* and *CYP71A4* genes were only separated by approximately 7.6 kb. Both genes also sit in a cluster on a separate chromosome from the cluster where UDT C6 and p-coumaryl CoA *2'hydroxylase* genes, were located. Genes transcribing the enzymes involved upstream of the furocoumarin biosynthesis pathway were seen to cluster together with both UDT C6 and p-coumaryl *CoA* genes, while the other *P450* genes were clustered together with *CYP71A3* and *CYP71A4* genes (Roselli et al., 2017). This study goes against the common idea that genes from a similar self-defence pathway would be found together as an evolutionary advantage. It could be possible that further downstream genes can be found on a separate cluster or anywhere separately on the genome. Further studies still need to be done to fully understand the biosynthesis of psoralen and angelicin including its location on the genome and the co-localization within the plant cell.

## Anti-Cancer and Anti-Tumor Properties

In the year 2013, cancer claimed the lives of 8.2 million people worldwide, making it the world's second leading cause of death, second only to cardiovascular diseases. In 2013 alone, there were 14.9 million new cases of cancer reported (Global Burden of Disease Cancer Collaboration, 2015). For women, breast cancer remains the most prevalent cancer, while for males, lung cancer tops the chart in developed and developing countries. Prostate cancer is also on the rise for men at the global level; and cervical cancer shows a similar trend among women (Global Burden of Disease Cancer Collaboration, 2015; Torre et al., 2015). Colorectal, stomach, and liver cancer are the next most frequently diagnosed cancers globally. Many factors are believed to contribute to the increased incidence of cancer including lifestyle behaviors which have become more common over the decades such as smoking, physical inactivity, poor diet and later dates of first births—all of which have been suggested to increase the risk of cancer (Torre et al., 2015).

The basic hallmarks of cancer include its ability to evade the immune system, resist cell death and growth suppressors, maintain cell proliferation, induce angiogenesis as well as activate invasion and metastasis (Hanahan and Weinberg, 2011). These properties make cancer a difficult disease to manage and eradicate. However, by targeting apoptotic pathways, such as the AKT pathway, MAPK pathway, and modulating the expression of pro- and anti-apoptotic proteins, anti-cancer drugs can slow the progress and even reduce the spread of cancer (Wong, 2009; Falasca, 2010). Another key factor for a good anti-cancer drug is that the drug should have as specific as possible cytotoxicity toward cancer cells only while sparing healthy cells as much as possible. Therefore, natural products have become the research focus as they are easily obtained, safer to use and have low toxicity (Pratheeshkumar et al., 2012).

Many studies have been conducted to test for anti-cancer properties in angelicin, with several of these demonstrating angelicin's ability to reduce the cell viability of human prostate cancer (PC-3), human epithelioma (Hep2), colorectal carcinoma (HCT116), rhabdomyosarcoma (RD), human cervical carcinoma (HeLa) cell line, cervical squamous cell carcinoma (SiHa) cell line and human breast adenocarcinoma (MCF7) cell lines (Mira and Shimizu, 2015; Wang et al., 2015a; Wang et al., 2019b). Historically, the first cell line tested which showed angelicin's anti-cancer properties was the neuroblastoma (SH-SY5Y) cell line, which are cells derived from a metastatic bone tumor biopsy. It was found that when treated with angelicin, upregulation of both caspase 3 and 9 could be seen. In addition, anti-apoptotic proteins were also affected by angelicin in SH-SY5Y cells. Bcl-2, Bcl-xL, and Mcl-1 proteins were seen to decrease their expression levels together with procaspase 9 (Rahman et al., 2012; Kovalevich and Langford, 2013). Irregularity in the regulation of the anti-apoptotic proteins is one of the factors that contribute to the development of cancer as overexpression of them blocks apoptosis and makes the cells resistant to anti-cancer drugs (Oltersdorf et al., 2005). When decreased in its expression, cancer cells became less resistant to anti-cancer therapies (Webb et al., 1997). Hence, the changes seen in the expression of apoptotic and anti-apoptotic proteins by angelicin is a positive indicator that angelicin has anti-cancer properties. Besides that, several different cells, such as promyelocytic leukaemia (HL-60), human lung cancer (A549), hepatoblastoma (HepG2), and hepatocellular carcinoma (Huh-7) cell lines, also yielded the same changes in the proteins mentioned above when incubated with angelicin for 24 or 48 h (Yuan et al., 2015; Li et al., 2016; Wang et al., 2017a). This shows that angelicin has the potential to be effective against multiple cancer cell lines. Besides seeing a decreased expression of anti-apoptotic Bcl-2 family proteins, increased expression of apoptosis-inducing Bcl-2 family, Bax, was also reported in the treated cell lines. Even the expression levels of cytochrome C was increased dose-dependently when tested in HepG2 and Huh-7 cells (Wang et al., 2017a). An increase in the expression of cytochrome C and Bax had been known to indicate the activation of the intrinsic apoptotic pathway. This is because Bax protein causes the permeation of the mitochondrial outer cell membrane, thus releasing cytochrome *c* into the cytosol, which then triggers the caspase 3 and 9 cascades, mediating apoptotic programmed cell death (Fulda and Debatin, 2006; Ashkenazi, 2008). Hence, based on what had been reported, it is possible that angelicin mainly induces cell death *via* the intrinsic apoptotic pathway in various cancer cell lines.

Having demonstrated possible activity in inducing apoptosis *via* the intrinsic pathway, the possibility of angelicin to have effects on the extrinsic pathway was also studied. In human SH-SY5Y cells no changes in the regulation of FAS receptor, FAS ligand and caspase 8 were seen, suggesting that the FAS pathway may not be activated by angelicin (Rahman et al., 2012). On the contrary, human renal carcinoma (Caki) cells displayed a different kind of result when incubated with angelicin. Caki cells have always been known to be one of those cancer cell lines that are resistant to tumor necrosis factor (TNF) apoptosis inducing ligand (TRAIL) (Han et al., 2016). When the compound was tested alone, angelicin was not able to induce apoptosis, yet when incubated together with 50 ng/ml of TRAIL the combined treatment was able to promote cell death (Min et al., 2018). Further investigation then went on to show how this combination treatment was even more effective than cycloheximide in downregulating cellular FLICE (FADD-like IL-1β- converting enzyme)-inhibiting protein (c-FLIP) post-translationally. Active caspase 3 was also upregulated and poly (ADP-ribose) polymerase was also cleaved, confirming cell apoptosis, though the mechanism was independent of endoplasmic reticulum (ER) stress and reactive oxygen species (ROS) signaling. Also, there were no changes with death receptor 5 (DR5) as well as the other intrinsic related apoptosis proteins, which suggest that angelicin does not induce apoptosis through the intrinsic pathway or DR5 in the Caki cells. There were also no changes in the inhibitor of apoptosis proteins (IAP) family, which includes cIAP1, XIAP, and survivin. Interestingly, the pro-apoptotic protein Bim was shown to increase in its expression when Caki cells which were only treated with angelicin; however, combination treatment with TRAIL attenuated the effect. The combination treatment of angelicin and TRAIL was able to induce apoptosis in other cancer cell lines, such as Sk-hep1 and MDA-MB-361 cells but normal cell lines were not affected (Min et al., 2018). This is a good therapeutic option to be exploited as this combination treatment could have the potential to be used as targeted cancer therapy toward cancer cell lines that are resistant to TRAIL.

In many cancer cell lines, the PI3K/AKT signaling pathways are antagonized by a mutation or deletion on its tumor suppressor gene, phosphatase and tensin homolog (PTEN). This mutation then leads to an attenuation of the intrinsic apoptosis pathway causing the cells to increase in cell growth and proliferation, become anti-apoptotic and increase in their angiogenesis and metastasis abilities (Downward, 2004; Roy et al., 2010). To further study the effect angelicin on the PI3K/AKT, Wang et al. (2017a) treated two liver cancer cell lines, HEPG2 and Huh-7 with angelicin. The results showed a significant reduction in the expression of PI3K and phospho-AKT proteins in both cell lines in a dose-dependent manner. However, Li et al. (2016) and Rahman et al. (2012) reported no changes in the PI3K/AKT pathway were seen in A549 lung cancer cells and SH-SY5Y neuroblastoma cells when treated with angelicin. Pro-apoptotic GSK-3β also did not experience any changes in the neuroblastoma cell line (Rahman et al., 2012). From this observation, it is possible that angelicin may be affecting the upstream processes such as the promoter or silencer genes in this pathway and not the protein expression itself. It could be a different mutation in these upstream genes in both A549 and SH-SY5Y cells that prevents angelicin from affecting the PI3K/AKT pathway in both these cell lines.

The mitogen-activated protein kinase (MAPK) signaling pathway has also been investigated as it is involved in cell proliferation or cell death. In A549 cells, an increase of phosphorylated JNK (pJNK) and phosphorylated ERK1/2 (pERK1/2) was seen but no changes in p38 MAPK was observed (Li et al., 2016). Again, this was different for SH-SY5Y cells in which the MAPK pathway (including p-38, pERK 1/2 and pJNK) was not affected (Rahman et al., 2012). The phosphorylation of JNK had been associated with the upregulation of pro-apoptotic genes while phosphorylation of ERK 1/2 has often been related to increased cell proliferation and survival. However, there had been a discussion that ERK1/2 may also be involved in apoptosis although its death mechanism has yet to be fully understood (Mebratu and Tesfaigzi, 2009; Wagner and Nebreda, 2009). In this case, inhibition of both ERK and JNK reduces the apoptotic effect which suggests the significant role of MAPKs in angelicin-mediated apoptosis in cancer cells (Li et al., 2016).

Other researchers have also investigated different aspects of angelicin in inhibiting cancer proliferation. A study on cell cycle arrest showed that angelicin downregulates cyclin B1, cyclin E1 and Cdc2 in human lung cancer A549 cells. The cell cycle assay using flow cytometry also showed an increase in cell cycle arrest at the G2/M phase and a decrease in G0/G1phase (Li et al., 2016). However, angelicin induced cell cycle arrest might differ in different cell lines. For example, HeLa and SiHa cells were significantly arrested at the G0/G1 phase while the ratio of cells was significantly decreased in the G2/M phase (Wang et al., 2019b). This could perhaps be due to the different pathways that are affected by angelicin in different cell lines. Apart from that, angelicin was also able to inhibit tubulin polymerization; in fact, it was able to inhibit this to a higher degree than psoralen according to the rank score match of both compounds with the colchicine binding residue on microtubules and histone deacetylase 8 inhibitory (HDAC8) assay (Mira and Shimizu, 2015). This is certainly an interesting area to look into as inhibition of microtubules can affect the cell proliferation of cancer cells (Jordan and Wilson, 2004). Angelicin had also been tested for its ability to reverse multidrug resistance in resistant human myelogenous leukemia (K562/A02) cell line but the results were not significant (Wang et al., 2016).

Another aspect that was looked into was the effect of angelicin on autophagy in cancer cell lines. According to Wang et al. (2019b), angelicin's anti-cancer properties on HeLa and SiHa cells could be due to its ability to inhibit autophagy. Autophagy is a process where molecular materials and organelles of cells are degraded in autophagosomes, aided by lysosomes (Maiuri et al., 2007). For cancer cells, autophagy was suggested to function in prolonging its survival by mitigating the effects of cellular and environmental stresses (Mathew et al., 2007). This catabolic process of autophagy is regulated by the mammalian target of rapamycin (mTOR) signaling pathway (Kim and Guan, 2015). It had been reported that the negative regulation of the mTOR pathway induces autophagy when cells are exposed to nitrosative and oxidative stress (Tripathi et al., 2013; Tang et al., 2014). In autophagy, the microtubule-associated protein 1 light chain 3 (LC3) was initially converted into LC3-I (a cytosolic form of LC3) and then LC3-phosphatidylethanolamine conjugate (LC3-II) when autophagosomal membranes were formed. LC3-II is then localized onto autophagosomes or autophysosomes (fusion of autophagosome and lysosome) (Tanida et al., 2005). From the study done on HeLa cells and SiHa, it can be seen that angelicin not only increased the phosphorylation of mTOR protein but also decreased the expression of LC3B-II in both cell lines (Wang et al., 2019b). Other than that, the expression of Atg3, Atg7, Atg12-5, which are essential in the formation of autophagosomes, were decreased as well (Wang et al., 2019b). Based on the data that was obtained, this indicates the involvement of angelicin in the autophagy process of cancer cells.

As described above, many different cell types from neurological to liver cell lines have been used to study the mechanism of angelicin's anti-cancer properties. Yet, testing the effectiveness of angelicin's anti-cancer properties against cells lines could not fully emulate actual *in vivo* situations in the human body. Therefore, further tests on animal models would help us understand if similar effects seen on the cell lines would be applicable in an organism. One study treated UMR-106 (rat osteosarcoma) cell lines that were injected in the tuberosity region of the tibia in nude rats, with 320 and 1,600 μg/kg/d angelicin for 10 days (Lu et al., 2014). After the rats were sacrificed, the osteosarcoma volume and weight in treated rats experienced a significant decrease, as compared to the control. Besides that, there were also hallmarks of cell death such as an increased amount of necrotic tumor cells, cell debris, shrunken nuclei, condensed cells, tissue haemorrhaging, aggregation of inflammation, and infiltration of lymphocytes. On the other hand, the liver, spleen, kidney, heart, and lungs showed no changes after treatment. There were also no metastatic lesions in the abdomen (Lu et al., 2014). The decrease of alkaline phosphatase (ALP), an essential factor in bone formation, in the serum also indicated that angelicin was able to reduce osteosarcoma cell growth *in vivo*, showing that angelicin decreased osteosarcoma tumor growth (Lu et al., 2014). Another study by Li et al. (2016) on A549 cancer cell lines, involved implanting A549 cells in the right flanks of female nude mice. These mice were then treated with angelicin orally for 4 consecutive weeks. After the 4-week treatment, the mice were sacrificed and the tumor was retrieved for further study. It was found that the weight and the size of the tumor had been considerably reduced. In addition, the tumor also showed decreased matrix metalloproteinase 2 (MMP2) and matrix metalloproteinase 9 (MMP9) expression levels while there were increased E-cadherin levels. This indicates that angelicin was able to have an inhibitory effect on tumor metastasis. The results on the expression levels of these three proteins correlate with those obtained from the cell line, including the experiment on wound healing that was done to show the inhibition of angelicin on cell migration (Li et al., 2016). A similar decrease in migration and invasion were also reported in *in vitro* model of HeLa and SiHa cells by Wang et al. (2019b), indicating that such inhibition was not limited to A549 cell lines only but also that angelicin is able to demonstrate anti-invasion and anti-migration properties in both *in vitro* and *in vivo* models. Wang et al. (2017a) discovered that angular furocoumarin decreases the growth of HepG2-induced tumor that was implanted in the back of male BALB/c mice. At the same time, the levels of proliferation marker Ki-67 and phosphorylated vascular endothelial growth factor 2 (p-VEGFR2) were significantly reduced in the tumor tissues. From this observation, it could be suggested that angelicin may be suppressing the proliferation of the tumor by disruption of vascularity, thus suppressing angiogenesis. No reduction of body weight and cases of mortality were reported, indicating the selectivity of angelicin toward cancer cells (Wang et al., 2017a).

Recently, a report depicting molecular docking analysis of angelicin on breast cancer markers was published. In breast cancer, the estrogen receptor (ERα) levels were highly expressed and its presence maintains the survival and growth of breast cancer cells (Fillmore et al., 2010). Another study on estrogen-progestin replacement therapy suggests that this regimen can be associated with an increased risk of breast cancer (Schairer et al., 2000). Epidermal growth factor receptor (EGFR) is also an important marker to look at in cancer, especially breast cancer, as it can affect the differentiation and proliferation of cancer cells (Tsutsui et al., 2002). Hence, to be able to inhibit these breast cancer marker proteins would aid in the control or decrease of breast cancer. As Acharya et al. (2019) had reported using molecular docking, angelicin displayed low binding energy toward ERα (-12.01 kcal/mol), progesterone (PR) (-11.63kcal/mol), EGFR (-12.6kcal/mol) and mTOR (-13.64kcal/mol). When compared to psoralen, the binding energy for ERα was even lower in angelicin. To confirm the ability of angelicin were able to bind to the protein of interest, *in vitro* analysis was done on breast cancer (MCF-7) cell line for the analysis of EGFR while the binding of angelicin to ERα was measured using ERα reporter assay system. Both the data obtained showed that angelicin was able to act as an antagonist for both proteins. For ERα, when comparing the IC_50_ value between angelicin and psoralen for antagonizing ERα, the IC_50_ value for angelicin was much lower, signifying that angelicin could antagonize ERα better than psoralen. However, both angelicin and psoralen were unable to inhibit mTOR (Acharya et al., 2019). Nevertheless, angelicin displayed potential as an antagonist against breast cancer cells.

**Figure 5:**
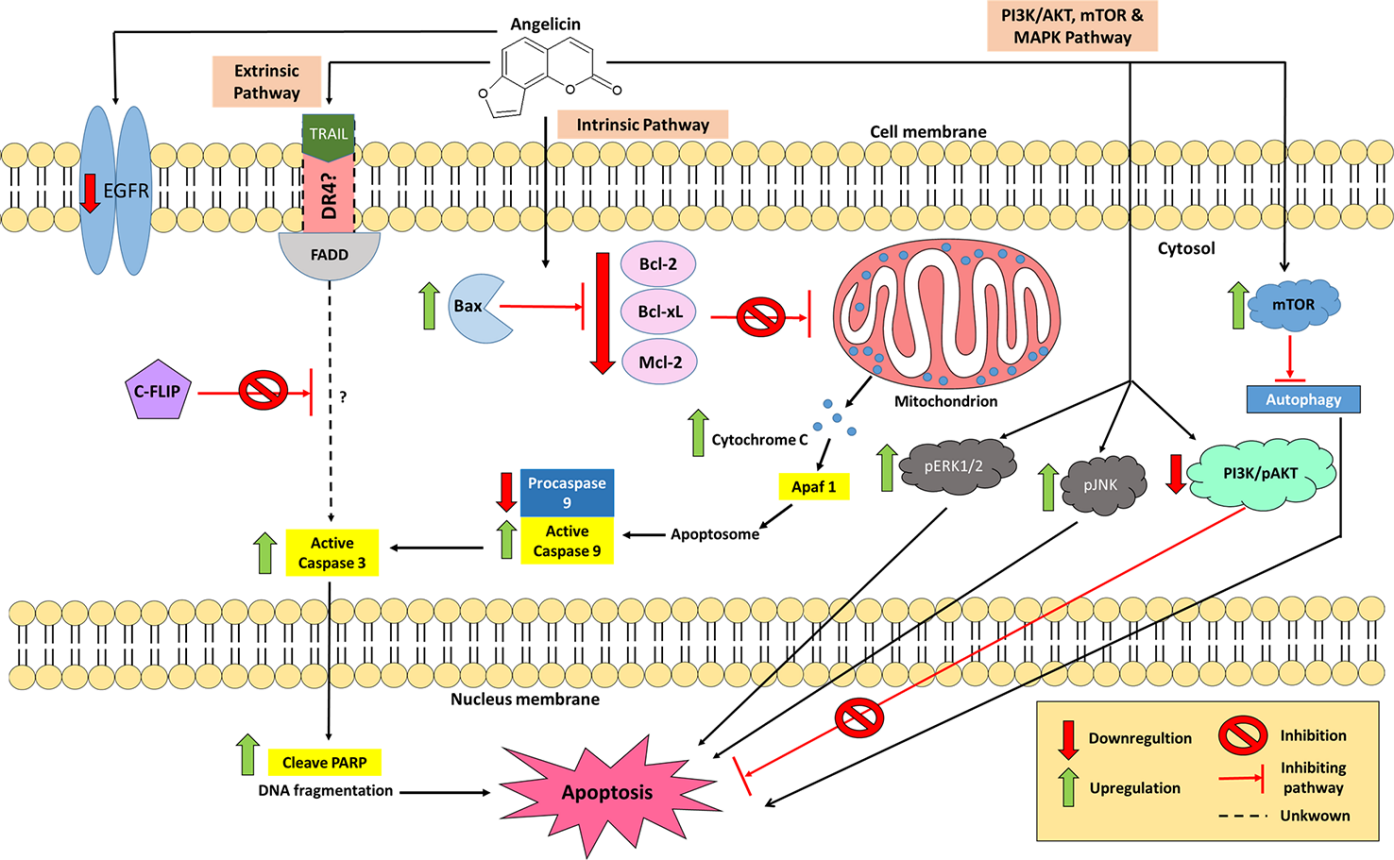
The involvement of angelicin in several apoptotic pathways which promote cancer cell death. In the intrinsic pathway, angelicin increases the expression of proapoptotic proteins and decreases the expression of anti-apoptotic proteins, causing a caspase cascade of caspase 3 and 9 to occur. In the extrinsic pathway, combination treatment between TRAIL and angelicin down-regulated c-FLIP which leads to an increase in active caspase 3, inducing cell apoptosis. However, the involvement of other apoptosis-related proteins with the combination treatment in the extrinsic pathway is yet to be elucidated. The PI3K/AKT and MAPK pathways are also actively involved in angelicin-mediated cancer cell death.

Through the studies shown, angelicin has great anti-cancer potential due to its specific cytotoxicity toward cancer cell lines both *in vitro* and *in vivo*. This is a much better alternative compared to psoralen which was shown to be more cytotoxic toward normal cells than angelicin. Angelicin also exhibits positive cytotoxicity toward multiple cancer cell lines *via* the intrinsic as well as extrinsic apoptotic pathways. In some cell lines, the involvement of MAPK, mTOR, and PI3K/AKT pathways in the action of angelicin casts some light not only on its apoptotic effect but also indicates the compound's ability to interfere with the survival of cancer cells through other mechanisms. Other than that, the recent study using molecular docking to predict the inhibitory activity of angelicin against specific cancer markers is promising for a whole new avenue in producing targeted cancer drugs. A summary of the different pathways that are involved in angelicin-mediated apoptosis in cancer cells is depicted in **Figure 5**.

## Anti-Inflammation Properties

Inflammation is a response of the body toward tissue injury, infection or irritants. It is mainly caused by non-specific and specific immune responses to limit the spread of pathogens or injury (Watkins et al., 1995). Inflammation can be caused by many factors for example open cut or internal wounds, oxidative stress, viral and bacterial infection. These factors can cause either acute or chronic inflammation. Acute inflammation is a type of inflammation that persists for a short time and is advantageous to the host but chronic inflammation lasts for an extended period of time and when not suppressed, can cause damage to normal cells and excessive tissue damage (Rahman and MacNee, 2000; Reuter et al., 2010). The activation of NF-κB is one of the inflammatory responses toward stressors like physical stress, physiological stress, pathological invasion, and environmental stress (Liu and Malik, 2006). Once stimulated, the NF-κB dimer is released from its inhibitor IκB and it then enters the nucleus to initiate the transcription of genes that are involved in the complex regulation of inflammatory mediator networks and cytokines(Liu and Malik, 2006; Rai et al., 2015). This then initiates the recruitment of inflammatory cells like neutrophils to the inflamed site to release anti-microbial peptides and ROS to kill invading microbial pathogens and initiate cell death when necessary. However, overproduction of ROS could also lead to extensive tissue damage which is dangerous for the host (Yamamoto et al., 2008; Morgan and Liu, 2011).

Through mimicry of acute lung injury *via* lipopolysaccharide (LPS) induced inflammation of murine lungs, Liu et al. (2013) showed that pre-treatment with angelicin reduces the production of pro-inflammatory cytokine markers, such as IL-6 and TNF-α. Not only that, the accumulation of polymorphonuclear neutrophils and macrophages in the lungs was also reduced by the pre-treatment. Histological images of the lung tissue also displayed reduced hallmarks of lung damage such as alveolar haemorrhage, alveolar wall thickening, interstitial edema, and infiltration of inflammatory cells. A deeper look into the mechanism behind angelicin's anti-inflammatory properties revealed that angelicin actively blocked the phosphorylation of IκBα and p65 in the NF-κB pathway, blocking the translocation of NF-κB into the cell nucleus.

Additionally, angelicin also showed inhibition of phosphorylation of p38 and JNK pathways in MAPK which decreases sepsis-induced organ injury within the mice. However, the ERK pathway, which is involved in cell growth and proliferation, was not inhibited by angelicin (Liu et al., 2013). Similar findings had been discovered in other studies on inflammation in asthmatic mouse models. Treatment with angelicin in asthmatic mice inhibits the production of cytokines IL-4, 5, and 13 that are typical of inflammation in asthmatic patients. With the inhibition of these interleukins, the production of serum IgE and airway hyperresponsiveness were also inhibited. The researchers also recorded similar NF-κB pathway inhibition, as evidenced by a significant decrease in the phosphorylation of p-65 and IκB (Wei et al., 2016). This suggests that it is possible that the anti-inflammatory properties of angelicin involve the inhibition of NF-κB pathway in inflammatory- related respiratory ailments. The inhibition then, in turn, stops the production of cytokines necessary to initiate the inflammatory reaction.

Besides that, the anti-inflammatory properties of angelicin were also tested on mouse neuronal cells. Inflammation of the brain is known to be associated with many neurodegenerative diseases such as Parkinson's, Alzheimer's, Huntington's disease and stroke (Coombes et al., 2011; Nirmaladevi et al., 2014; Yang et al., 2016). For example, Alzheimer's patients have higher levels of ROS, produced partly due to inflammation, as compared to a normal person (Yang et al., 2016). Reactive nitrogen species (RNS) such as nitric oxide is also produced excessively by activated microglia and astrocytes which is deleterious toward the neurons (Liu et al., 2002). Angelicin was able to exert a neuroprotective effect on neural cells by inhibiting the occurrence of inflammation. When angelicin was applied to LPS-induced inflamed mouse BV2 microglia cells, nitric oxide (NO) production of the cells was significantly suppressed. This is a complete contrast to psoralen which showed no changes in the production of NO, making angelicin an interesting subject for further study. Other treatments involving the application of a non-radical species of ROS, hydrogen peroxide (H_2_O_2_), toward HT22 mouse hippocampal cells to initiate neuronal cell damage were also attenuated by angelicin. Again, the result is very different for psoralen, which was only able to slightly reverse the neuronal cell damage caused by H_2_O_2_ (Simon et al., 2000; Kim et al., 2016). With the results obtained from this study on neuronal cells, angelicin appears to be a good candidate for further analysis in human models to study its mechanism in neurodegenerative diseases.

On another note, a study done by Chen et al. (2017), showed that when angelicin was tested against human neutrophils obtained from the blood, the inhibitory effects of angelicin against the generation of superoxide radical anion and the release of elastase were shown to be less effective as compared to its sister isomer, psoralen. A weaker inhibitory effect was also seen from angelicin against the production of NO from RAW264.7 murine macrophages as compared to psoralen. The results obtained were the opposite of what was seen as described by Kim et al. (2016). It is possible that the pathways activated by angelicin in neuronal cells are different from those of human neutrophils. It should also be noted that in the study done by Liu et al. (2013) the NO production of RAW 264.7 mouse macrophage cell line was not tested. Hence, a deeper understanding of the pathways activated by angelicin and psoralen is still very much needed.

**Figure 6:**
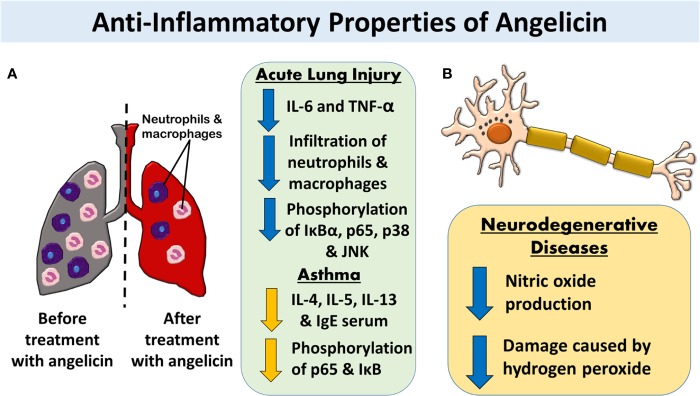
The anti-inflammatory properties of angelicin. **(A)** Angelicin attenuates inflammation-induced-damage in acute lung injuries and asthma by lowering the cytokine production and reducing the infiltration of neutrophils and macrophages. Both MAPK and NF-κB pathway was also affected by angelicin in which angelicin inhibits the phosphorylation of IκB, p65, p38, and JNK. **(B)** Angelicin exerts a neuroprotective effect by inhibiting the production of nitric oxide and reducing the damage caused by hydrogen peroxide in LPS-induced inflamed mouse BV2 microglia cells and HT22 mouse hippocampal cells respectively.

Nevertheless, angelicin had shown great promise in its anti-inflammatory aptitude especially as a neuroprotectant and against inflammatory-related respiratory ailments as shown in **Figure 6**. Perhaps with chemical modification of angelicin and a greater amount of research on the pathways involved, it is possible that angelicin can be used in developing a new anti-inflammatory drug.

## Angelicin as an Osteogenesis and Chondrogenesis Enhancer

Bone remodeling is a process that is coordinated by bone resorption and bone formation, mediated by osteoclast and osteoblasts respectively (Tang et al., 2009). In bone formation, transforming growth factor (TGF-β) and bone morphogenetic proteins (BMPs) are key initiators of osteogenic differentiation *via* the TGF-β/BMP signaling pathway (Chen et al., 2012). At the start of signaling, BMP/TGF-β interacts with either BMP or TGF-β specific type 1 and 2 serine/threonine kinase receptor to initiate BMP/TGF-β-regulated cascade through canonical (Smad dependent) and non-canonical (smad independent) pathways. In the canonical pathways, smad 1, 2, 3, and 5 are activated by BMP and TGF-β extracellular signals, while smad 4 forms complexes with the other smads and translocate into the nucleus, initiating transcription of selected genes such as Runx2/Cbfa1/Osf2/AML3. Other smads such as smad 6 and 7 act as negative regulators of BMP/TGF-β signaling pathway (Gersbach et al., 2004; Hayrapetyan et al., 2014). Runt transcription factor 2 (Runx2), is an essential transcriptional activator in osteoblastic differentiation (Gersbach et al., 2004). Runx2 can also be induced by Wnt/β-catenin signaling. In an experiment done by Gaur et al. (2005) it was seen that when Wnt proteins were upregulated in secreted fizzled -related protein-1 (SFRP1) null mice, the expression of Runx2 mRNA and protein levels were significantly increased up to 6–9 fold and 2–3 fold respectively. The increase in Runx2 was also paralleled with an increase in osteocalcin (OCN) by 1.3–3 fold higher than wild type mice. This suggests a positive involvement of Wnt/β-catenin signaling in the expression of Runx2 and its contribution to osteogenic differentiation.

In both the primary bone marrow mesenchymal stem cells (BMSC) (obtained from 4 week old C57BL/6 mice) as well as primary rat osteoblasts cells (obtained from female Winstar rats) that were treated with angelicin, significant increases in BMP-2, Runx2, OCN, and β -catenin were reported in multiple studies (Wang et al., 2017b; Ge et al., 2019). Using immunofluorescence staining, it was also revealed that not only was β-catenin upregulated by angelicin, there was also an increase in β-catenin translocation into the nucleus after treatment (Ge et al., 2019). To determine the involvement of Wnt/β-catenin signaling pathway, the cells were co-treated with both angelicin and DKK-1, an antagonist to the pathway. The results obtained revealed that DKK-1 successfully attenuated the effect angelicin had on the cells (Ge et al., 2019). Besides that, Zhang and Ta (2017) also studied the effect of angelicin on TGF-β1 reporter gene activity using transfected HEK293T cells. The cells were firstly transfected with (CAGA)12-Luc-reporter plasmid and then stimulated with various concentrations of angelicin for 12 h before luciferase activity was measured. After the measurement, the results obtained showed that angelicin significantly increased the luciferase activity and thus the activation of TGF-β1 reporter gene. However, the mechanism behind the activation of this reporter gene was not studied. Other than that, in MC3T3-E1 (mouse osteoblast precursor) cell line, the osteogenic differentiation inhibitor smad 7 protein expression were downregulated, while the expression of COL1A1 mRNA, which encodes the type I procollagen chain, and collagen type I (COL-1) protein were upregulated by angelicin as well (Zhang and Ta, 2017; Ge et al., 2018). COL-1 is one of the major organic components found in the bone extracellular matrix (Lynch et al., 1995). When rat bone marrow cells were cultured together with COL-1 matrix gel, the cells exhibited signs of osteoblastic differentiation such as the formation of nodules and expressed osteoblastic specific genes like OCN, osteopontin (OPN) and bone sialoprotein (BSP), indicating its involvement in osteogenic differentiation (Mizuno et al., 2000). Hence, the increase in expression of COL-1 protein, suggests that angelicin is able to induce osteogenic differentiation in osteoblast cells.

To demonstrate and confirm further the pro-osteogenic effect of angelicin, the ALP activity and mineralization of osteoblasts were also measured. ALP is a metalloenzyme that has an essential role in the mineralization and is often used as an indicator of early-stage osteoblastic differentiation (Golub and Boesze-Battaglia, 2007). Mineralization occurs when a calcium phosphate known as hydroxyapatite is deposited in the extracellular matrix by either chondrocytes in growth plate cartilages, osteoblasts in bones or odontoblasts in the teeth *via* matrix vesicles (Orimo, 2010). As hydroxyapatite crystals propagate beyond the matrix vesicle into the spaces between the collagen fibrils, the ratio of inorganic phosphate (Pi) and inorganic pyrophosphate (PPi), an inhibitor of hydroxyapatite formation, must be managed. Therefore, the role of ALP in the generation of Pi by hydrolyzing PPi is essential for osteogenic differentiation (Orimo, 2010). In the year 2014, when angelicin was tested against primary rat osteoblastic cells obtained from newborn Winstar rats, it was initially reported that angelicin had no effect on cell proliferation or alkaline phosphatase (ALP) activity (Li et al., 2014). However, in a recent paper, Ge et al. (2019) had demonstrated that angelicin was able to not only increase the proliferation of osteoblast but also to induce the expression of alkaline phosphatase (ALP) after 7 days of treatment while the matrix mineralization of the cells can be seen on the 12^th^ day of treatment with angelicin. Similar observation of increased cell proliferation, ALP activity and mineralization of osteoblasts by angelicin were reported in MC3T3-E1 (mouse osteoblast precursor) cells and primary bone marrow mesenchymal stem cells (BMSC) with the exception that BMSC cells did not experience any changes in cell proliferation (Wang et al., 2017b; Ge et al., 2018).

Other than that, angelicin was also discovered to suppress the conversion of BMSC to adipocytes. The study by Wang et al. (2017b), reported an angelicin induced dose-dependent downregulation in PPARγ and C/EBPβ adipocyte-specific markers in adipogenesis differentiation while osteogenic markers, Runx2 and OCN were dose-dependently increased in osteogenic differentiation. In addition, two markers from the mTOR complex 1 (mTORC1) signaling pathway, p-S6 and p-4EBP1, were analyzed in both osteogenic and adipogenic conditions. When comparing the two markers, p-S6 showed marked downregulation in expression while p-4EBP1 was upregulated under both differentiation conditions for 7 and 14 days (Wang et al., 2017b). mTOR is a conserved pathway that plays a role in regulating cell growth and metabolism and it had been reported that mTORC1 participates in adipogenesis (Polak et al., 2008; Betz and Hall, 2013). Mice that had whole-body knockout of S6 were reportedly more resistant to diet-induced obesity while whole-body knockout of 4E-BP1 exhibited the opposite results (Polak et al., 2008). Hence, with similar regulation of S6 kinase and 4EBP1 expression, this suggests that angelicin was able to suppress the adipogenic differentiation in BMSC *via* the mTOR pathway.

To mirror what was seen in *in vitro* studies, mice and rats were ovariectomized or orchidectomized to induce osteoporosis and then treated with angelicin to determine if angelicin was able to reverse the symptoms. In female C57BL/6 mice, there was an increase in trabecular thickness, bone volume/total volume and trabecular number in the distal femur after treatment with angelicin. This indicates an increase in bone mass by angelicin. Runx2 protein was also upregulated and PPARγ protein downregulated in the distal femur after treatment with angelicin. It can be suggested that the cells within the distal femur were going toward osteoblastic differentiation instead of adipocyte differentiation. To reconfirm the data, a decreased number of adipocytes in the bone marrow was obtained as well (Wang et al., 2017b). Another study on ICR mice was done as well, where mice were segregated based on gender and the difference of not only between osteoporosis model group and treated group was measured but also comparison was done between the different gender groups (Yuan et al., 2016). Although some of the markers that were measured between the non-osteoporosis group (control) and osteoporosis model group were not significant, it was interesting to note that there is some difference in the expression of these markers when comparing gender groups. Nevertheless, both groups did have an increase in bone strength, trabecular number, trabecular bone area, and intramembranous ossification after treatment with angelicin. The trabecular bone gap instead was decreased by angelicin although not as small as the control group (Yuan et al., 2016). Besides that, collagen type I degradation product (CTX-1), a marker that reflects bone and cartilage breakdown, in the serum of both genders were reported to have decreased with the treatment of angelicin (Landewé et al., 2006; Yuan et al., 2016). This shows that angelicin was able to reduce collagen degradation. The interesting differences between female and male mice after treatment were the osteogenic markers such as ALP, TRACP, and OCN. The ALP levels were reportedly increased in the serum of male mice when treated with 10mg/kg of angelicin while female mice did not display any changes. On the other TRACP was seen to decrease in female mice treated with 20mg/kg angelicin (Yuan et al., 2016). Despite this difference gender-wise for ALP and TRACP, when the data were measured in terms of ALP/TRACP both genders showed an increase in the ratio of ALP/TRACP, which suggests positive bone formation (Yuan et al., 2016). As for OCN, there were no changes in female mice after being treated or even when comparing between the control and osteoporosis model groups. Yet, in the male mice, osteoporosis model showed a significant increase in OCN expression in the serum but 10 mg/kg angelicin significantly reversed the expression (Yuan et al., 2016). It could be possible that the difference in expression of these markers could be due to different physiologies between both genders. Nevertheless, angelicin still proved to be effective in reducing osteoporosis symptoms in both males and females.

During bone resorption, the generation of ROS was also found to play an essential role. Increased ROS levels stimulate the activation and differentiation of osteoclasts *via* receptor activator of nuclear factor-κB (NF-κB) ligand (RANKL) which then binds to receptor activator of NF-κB (RANK) that was expressed on macrophages. This then leads to a cascade of events that ultimately form osteoclasts (Ha et al., 2004; Srinivasan et al., 2010). Osteoclasts themselves too generate ROS through tartrate-resistant acid phosphatase (TRACP). This is an enzyme that is expressed by osteoclasts and has two independent enzymatic activities. It not only produces ROS using Fenton's reaction at neutral pH but also functions as a phosphatase in acidic conditions (Halleen et al., 2003; Halleen et al., 2006). The formation of ROS aids in degrading type I collagen during bone resorption (Halleen et al., 1999). By using OB-6 osteoblastic cells as a subject of study, it had been discovered that angelicin was able to attenuate H_2_O_2_ induced damage in the osteoblasts (Li et al., 2019). The cells not only showed an increase in cell viability and a decrease in the apoptotic rate but also showed suppression of ROS generation with angelicin treatment. When measuring the cells' mitochondrial function, it can be seen that angelicin reversed the H_2_O_2_ induced downregulation of mitochondrial respiratory chain complex I (MRC-1), protecting the mitochondrial function in osteoblastic OB-6 cells. Furthermore, angelicin also successfully induced the osteogenesis differentiation *via* Wnt/β-catenin pathway even under oxidative stress. The increase in calcium accumulation, in osteogenic differentiation marker (OCN and Runx2) and Wnt/β-catenin pathway markers (β-catenin and tankyrase), were reported when angelicin was co- treated with H_2_O_2_ in OB-6 osteoblastic cells. In addition, *in vivo* studies on female Sprague Dawley rats showed similar results concerning angelicin's ability to attenuate oxidative stress. Antioxidants such as superoxide dismutase (SOD), glutathione (GSH), glutathione peroxidase (GSH-Px) activity in the blood were increased after the treatment with 10 and 20mg/kg angelicin while lipid peroxidation marker malondialdehyde (MDA) was reportedly reduced (Del Rio et al., 2005; Wang et al., 2018). Apart from that, apoptosis markers caspase3/9 were also downregulated in the bone of the rats, suggesting a suppression of cell apoptosis in the bone. The rats also demonstrated a decrease of ALP in the blood, when compared to the group that has osteoporosis, after being treated with 10 and 20mg/kg angelicin for 12 weeks. According to Atalay et al. (2012), postmenopausal women have much higher ALP levels in the blood as compared to those who are in the premenopausal stage due to the increased bone turnover rate in postmenopausal women. Hence, the decrease of ALP in the blood of the rats indicates that there is a decrease in bone turnover rate by angelicin. Further analysis of the calcium/creatine levels in the urine, calcium and leptin levels in the serum and measurement of the bone mineral density of the rats indicates positive osteogenic differentiation in the rats (Wang et al., 2018). Wnt and β-catenin proteins were also upregulated as well in the cartilage tissue which suggests the activation of Wnt/β-catenin pathway while downregulation PPARγ protein expression indicates suppression of adipogenesis (Wang et al., 2018). Angelicin also dose-dependently inhibited the upregulation of OCN, COL-1, and osteoprotegerin (OPN)—a soluble decoy receptor that inhibits the function and differentiation of osteoclasts—in the cartilage tissues of the rats with osteoporosis, bringing them back to almost normal levels as compared with the healthy rats (Udagawa et al., 2000; Wang et al., 2018). Based on the *in vitro* and *in vivo* analysis, angelicin may potentially be exhibiting pro-osteogenesis properties by inhibiting ROS in bone resorption.

Estrogen plays an important role in the formation of bones. In early menopausal women, those that were treated with medium and high doses of natural estrogen, 17β-estradiol, and estriol, for a year had shown signs of increased bone mineral content. The ALP in the serum of these patients too were decreased (Christiansen et al., 1982). Common drugs such as bisphosphonates, denosumab, strontium ranelate, and estrogen receptor modulators, are being used to increase the estrogen levels in menopausal women. However, there have been concerns over its side effects for long term usage (Rizzoli et al., 2011). Hence, potential compounds that could upregulate the body's estrogen levels are needed. The treatment of MC3T3-E1 cells and primary rat osteoblast cells with angelicin increased the expression of ERα protein (Ge et al., 2018; Ge et al., 2019). A similar increase in ERα by angelicin in the grey matter of ventral and dorsal horns were also reported in adult male C57BL/6 mice that had suffered spinal cord injury (Li et al., 2017a). Other than that, to determine if angelicin were only upregulating ERα and not ERβ, Xin et al. (2010) incubated HeLa cells with angelicin and they confirmed that angelicin only increases ERα transcription activity. Despite previously being reported by Acharya et al. (2019) that angelicin acts as an antagonist for ERα, the *in vitro* analysis done on osteoblasts and HeLa cells depicted the opposite reaction. Hence, various cell lines should be analysed to confirm the effect angelicin has on estrogen.

Furthermore, angelicin also was found to inhibit the nucleocytoplasmic shuttling of the aryl hydrocarbon receptor (AhR) from the cytoplasm into the nuclei by directly binding to the receptor itself. AhR acts as a negative regulator of osteogenic differentiation and instead promoted bone resorption. In AhR knockout mice, there was a decrease in bone resorption and an increase instead in bone mass. Osteoclasts were also inhibited from differentiating in AhR knockout mice (Yu et al., 2015). Hence, the inhibition of the AhR signaling pathway by angelicin could perhaps contribute to an even higher increase in osteogenic differentiation in osteoblast cells. Angelicin also inhibited the expression of cytochrome P450 family subfamily A member 1 (CYP1A1) in MC3T3-E1 cells (Yu et al., 2015). Not only that, when C57BL/c mice were injected with 10mg/kg angelicin and the expression of CYP1A1 in the serum decreased after treatment with angelicin (Ge et al., 2018). CYP1A1 is one of the genes that is mediated by AhR *via* the AhR signaling pathways (Delescluse et al., 2000). The CYP1A gene encodes for microsomal cytochrome P5401A1 protein, which catalyzes the metabolism of various xenobiotics (Qiang, 2001). Furocoumarins have been known to be an interfering agent in drug metabolism, especially with cytochrome P450 and this might pose a clinical advantage to increase the absorption of poorly absorbed drugs, thereby reducing dose requirements and costs of drugs (Girennavar et al., 2007). However, recent papers had expressed concern over its hepatotoxicity. Sprague Dawley rats that were treated with 60 mg/kg of angelicin and psoralen for 7 days experienced a change in the expression of 884 and 172 genes respectively with the metabolism of xenobiotics cytochrome P450 and chemical carcinogenesis pathway were the two most upregulated enriched pathway identified from the gene expression changes. The aspartate aminotransferase (AST), alanine aminotransferase (ALT), total bile acid (TBA), and total triglyceride (TG) levels in the serum were upregulated as well, which are signs of hepatotoxicity. In the cytochrome P450 pathway, CY1A1, CYP1A2, Gstm1, and Akr7a3 were predicted to be key genes in angelicin and psoralen hepatotoxicity. The endoplasmic reticulum too was predicted to play a role in angelicin and psoralen liver injury (Song et al., 2019). On the other hand, hepatotoxicity by angelicin was reported only in rats and not mice. A study on Winstar rats (fed with 40 and 80 mg/kg angelicin) and ICR mice (fed with 160 and 320 mg/kg) showed that the rats experienced hallmarks of cytotoxicity while the mice did not (Wang et al., 2019c). This shows that the hepatotoxicity of the liver by angelicin might occur in some species but not others. Nonetheless, care should be taken if angelicin were to be developed further into treatment.

In addition to studying osteoblasts, the effect of angelicin on chondrogenic differentiation was also analyzed. During the development of embryos, a process known as endochondral ossification forms bones of axial skeleton *via* a cartilage intermediate (Barry et al., 2001). It is during this process that mesenchymal cells condensate bi-potential chondro-osteoprogenitors in a pattern of future bones and chondrocytes differentiate to become cartilage templates. After proliferating and arranging themselves, the chondrocytes then differentiate into hypertrophic chondrocytes. These cells then secrete matrix vesicles which initiate cartilage calcification. Nearby bi-potential prechondrial cells also proceed to differentiate to osteoblasts and begin the formation of bones (Tsang et al., 2015). An essential regulator of chondrogenesis is SRY-box transcription factor 9 (SOX9). One of the many roles of SOX9 is to regulate the expression of collagen, such as collagen type II and X, and proteoglycan aggrecan in chondrogenic differentiation (Bell et al., 1997; Bi et al., 1999; Han and Lefebvre, 2008; Tsang et al., 2015). To determine that, pre-chondrogenic ATDC5 cells were treated with angelicin (Li et al., 2012). Despite no increase in cell growth rate being seen, the number of cartilage nodules, ALP and matrix proteoglycan were increased. The expression of chondrogenic differentiation marker genes such as SOX9, collagen II, collagen X, and BMP-2 were upregulated as well (Li et al., 2012). Even Smad 4, part of another independent pathway from SOX9, was induced (Li et al., 2012; Lim et al., 2015). Hence, it can be said that the TGF-β/BMP pathway might be involved in angelicin-induced chondrogenic differentiation *via* two separate paths, SOX9 and Smad4. Besides that, β-catenin and Runx2 were also reported to be upregulated together with OCN and BSP (Li et al., 2012). Although both β-catenin and Runx2 had previously been reported to be inhibited by SOX9, the expression of both these proteins might indicate the gradual shift from the initial proliferation and differentiation of chondroprogenitors toward the terminal differentiation of chondrogenesis, leading to cartilage matrix calcification and vascular invasion, and finally ossification (Goldring et al., 2006; Dy et al., 2012). According to Goldring et al. (2006), the expression of Runx2 increases at terminal differentiation and maintains its expression through the process of cartilage matrix calcification and vascular invasion, where β-catenin was then upregulated as well. This shows that the prechondrogenic ATDC5 is undergoing chondrogenic differentiation toward ossification under the influence of angelicin.

Other than studying the chondrogenic differentiation markers, the activation of well-known MAPK kinases (JNK, ERK, and p38) were studied as well. After treatment of the cells with angelicin, there was a marked increase in the phosphorylation of ERK and p38. However, there were no changes in the expression of pJNK. This shows that there is an activation of ERK and p38 by angelicin. Then to confirm that both these kinases are involved in angelicin induced chondrogenic differentiation, the kinases were inhibited using MEK/ERK and p38 inhibitors. With the inhibition of both these kinases separately, the number of cartilage nodules and ALP activity decreased even though the cells were co-treated with angelicin (Li et al., 2012). Hence, this indicates that angelicin mediates chondrogenic differentiation *via* the MAPK pathway.

**Figure 7:**
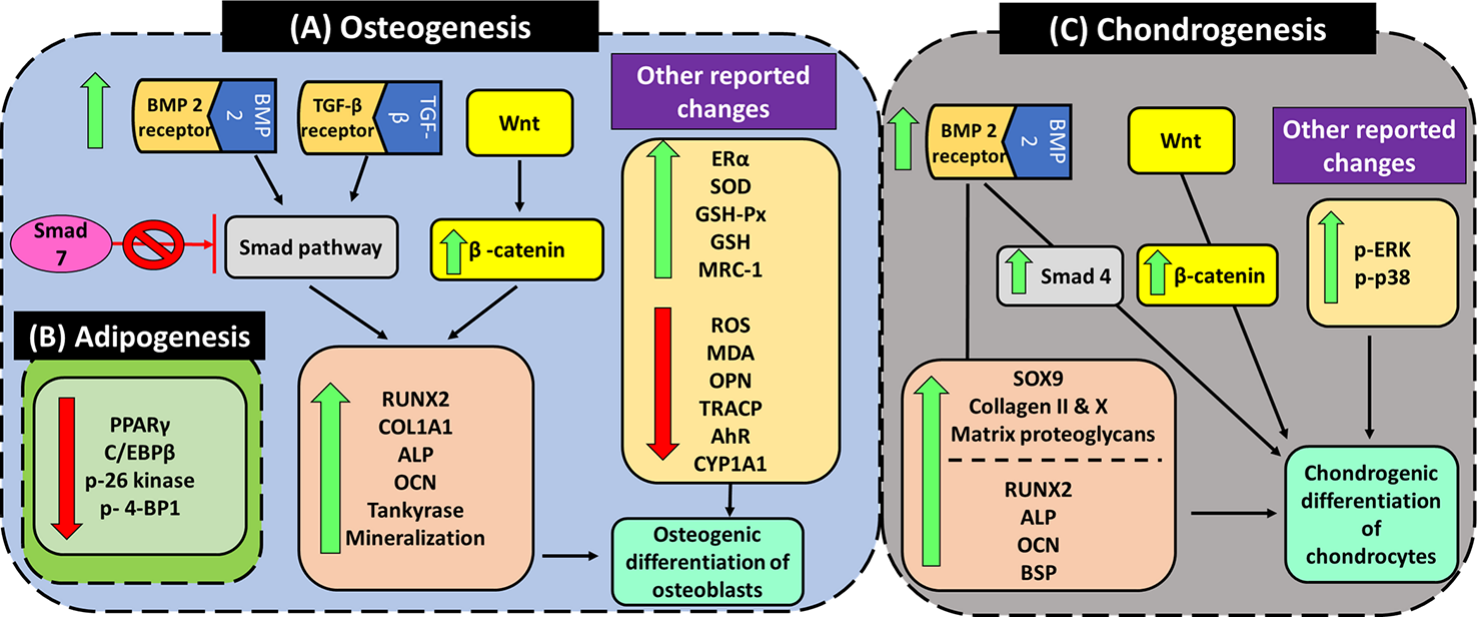
Angelicin exhibits pro-osteogenic, chondrogenic differentiating properties while suppressing adipogenic differentiation. **(A)** Several genes and proteins were upregulated by angelicin in the osteogenic differentiation *via* TGF-β/BMP, Wnt/β-catenin pathway and AhR pathway. It is also able to attenuate oxidative stress by upregulating antioxidant enzymes, inhibiting bone resorption. Other markers like ERα are upregulated as well by angelicin. **(B)** The suppression of adipogenesis by angelicin through the regulation of the mTOR pathway. **(C)** The induction of angelicin in chondrogenic differentiation *via* the Wnt/β-catenin, MAPK, and TGF-β/BMP pathway.

In summary, it can be suggested that angelicin not only induce osteogenic differentiation but also able to initiate chondrogenic differentiation. In this process, several pathways such as TGF-β/BMP, Wnt/β-catenin, AhR, and MAPK pathway were reported to be activated by angelicin. On the other hand, the suppression of adipogenesis, bone resorption gene markers and ROS by angelicin also aid in the increase osteogenic differentiation. A summary of the various pathways involved can be seen in **Figure 7**.

## Other Bioactivity Properties of Angelicin

In 2018, a new potential application for angelicin on periodontitis was investigated. This study not only involved the anti-inflammatory studies of angelicin but also explored its anti-microbial properties on *Porphyromonas gingivalis* (*P. gingivalis*). *P. gingivalis* is a gram-negative oral anaerobe that can induce inflammation in the periodontal tissue. This causes gum retrogression, bone weakness, and damage to the soft tissue. Advanced periodontitis can even cause tooth loss. What makes *P. gingivalis* unique is its ability to elude the immune response (Rafiei et al., 2017). In a study done by Li et al. (2018), angelicin was seen to be able to inhibit, reduce the formation and decrease the thickening of *P. gingivalis* biofilm. The minimal inhibitory and reduction concentration of angelicin against *P. gingivalis* biofilm was also surprisingly lower than psoralen. Besides that, angelicin was also able to decrease the viable counts of the bacteria either alone or in biofilm, while remaining non-cytotoxic toward human periodontal ligament cells (hPDLCs) and monocyte -like THP-1 cells. Angelicin was also able to significantly reduce the mRNA transcription and protein translation of inflammatory markers IL-1β and IL-8 in THP-1 cells while increasing osteogenic differentiation and the expression of osteogenic genes in hPDLCs cells (Li et al., 2018). This increase in the osteogenic differentiation and osteogenic genes expression in hPDLCs cells might also be due to angelicin itself as an osteogenesis promoter. *In vivo* tests on mice also mirrored results of the *in vitro* tests with histological images showing reduction of alveolar bone loss (Li et al., 2018).

Moreover, angelicin was reported to have anti-viral properties. In the research done by Coppey et al. (1979); Altamirano-Dimas et al. (1986) and Hudson and Towers (1988), angelicin had shown anti-viral activity against murine cytomegalovirus (MCMV),Sindbis virus (SV), bacteriophage T4, bacteriophage M13, and herpes simplex virus (HSV) under UVA exposure. Although its anti-viral activity level is not as strong as 8-MOP, it was suggested by Altamirano-Dimas et al. (1986) that the formation of monoadducts could still cause the viral genome to become non-infectious. Following these initial findings, angelicin was tested against the murine gammaherpesvirus 68 (MHV-68), which is biologically and genetically related to the human version. In this study, the replication of viral genome was decreased dose-dependently by angelicin as the compound inhibits the expression of replication and transcription (RTA) mRNA, which is responsible for the induction of the virus's lytic cycle, at the early stages of virus infection (Ragoczy and Miller, 1999; Cho et al., 2013). This, in turn, downregulates the expression of both tegument protein ORF45 and small capsid protein ORF65, both of which are late gene protein expression known to be trans-activated by RTA (Cho et al., 2013; Purushothaman et al., 2015; Wang et al., 2015b). When tested on plaque formation, angelicin was found to deter the formation of plaques after infection (post-treatment) but not before virus adoption. Although angelicin is not as effective as the positive control ganciclovir (GCV), which is a common anti-viral drug used against herpesviruses, it was much more effective as compared to psoralen. Further tests were also done on two famous strains of gammaherpesvirus; Kaposi's sarcoma-associated herpesvirus (KSHV) and Epstein-Barr virus (EBV). An inhibition by angelicin on the early stages of lytic replication was also experienced by both strains, similar to MHV-68. However, psoralen shows a better inhibition of KSHV than angelicin (Cho et al., 2013).

Besides that, the erythroid differentiation of angelicin was also investigated. Globally, 2 in 1000 individuals are afflicted with haemoglobinopathies and there is a frequency of over 4.5% of the human population who are carriers of this disease. Beta-thalassemia and sickle cell anaemia (SCA) are the most common blood disorders and are autosomal recessive diseases (De Rycke et al., 2001). To counter these blood disorders, the production of fetal haemoglobin (HbF) must be increased as it had been found that individuals with higher levels of HbF showed milder or alleviated symptoms (Stamatoyannopoulos et al., 1975; Uda et al., 2008; Xu et al., 2011). In an attempt to increase the HbF levels in SCA and thalassemia patients, drugs such as hydroxyurea, mithramycin and psoralen were used. These drugs were able to increase the HbF production by inducing an increase in γ-globin mRNA production with hydroxyurea being the most common drug to be used in high-income countries (Fibach et al., 2003; Dixit et al., 2005; Viola et al., 2008; Mulaku et al., 2013). In a study, angelicin displayed an ability to induce erythroid differentiation in K562 cells which was associated with the increase in the production of γ-globin mRNA. It was also noted that the ability of angelicin to stimulate γ-globin mRNA was higher than hydroxyurea, in the two-phase liquid cultural model, though it is not as strong as compared to mithamycin and cytosine arabinoside (Ara-C) (Lampronti et al., 2003). Another interesting study was also done on the photoproducts of angelicin which was obtained through pre-irradiation of angelicin before its application on K562 cells had shown that under anaerobic conditions, the photoproducts are also able to induce erythroid differentiation. Interestingly, in the two-phase liquid culture model, angelicin photoproducts showed even higher fold increases of adult haemoglobin (HbA) and HbF as compared to both 8-MOP and 5-MOP (Viola et al., 2008). This study is certainly intriguing and warrants further *in vitro* and *in vivo* studies not only on angelicin itself but also on its photoproducts.

**Figure 8:**
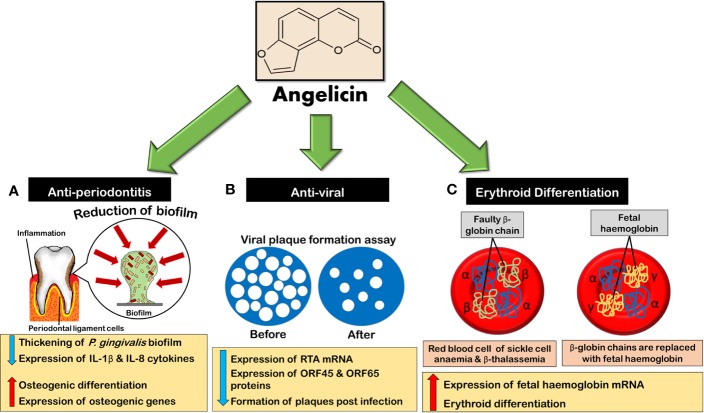
Angelicin exhibits anti-periodontitis, anti-viral and erythroid differentiating properties. **(A)** Angelicin reduces and inhibits the thickening of *P. gingivalis* biofilm while inhibiting the expression of pro-inflammatory cytokines. Besides that, angelicin also promotes osteogenic differentiation and an increase in the expression of osteogenic genes in hPDLCs. **(B)** Angelicin inhibits murine gammaherpesvirus 68 lytic cycle at early stages of infection by affecting the expression of RTA mRNA and thus indirectly the expression of ORF45 and ORF65 proteins, decreasing the formation of plaques. **(C)** Erythroid differentiation increases as angelicin and its photoproduct increase the expression of fetal haemoglobin mRNA.

To sum it up, more studies including *in vivo* studies are needed to be done on the anti-viral and erythroid differentiating properties of angelicin, hence to help us further understand their underlying mechanisms. Nevertheless, the study of angelicin on periodontitis also uncovered a new potential bioactivity, which is its anti-microbial properties. To date, only Li and colleagues had reported its anti-microbial activity. As angelicin is developed as part of a plant's self-defence mechanism, angelicin may possess anti-microbial activity not just only against *P. gingivalis* but also other pathogenic bacteria as well. Thus, research on its anti-microbial activity should also be looked into. A summary of angelicin's anti-periodontitis, anti-viral, and erythroid differentiating properties can be seen in **Figure 8**.

## Commercial Uses and Patents

According to “Scifinder”, not many patents for angelicin had been made. Out of 11,663 hits on “Scifinder” for the word “Angelicin”, only 59 were classified as patents. But even so, not more than 10 contain angelicin as the main ingredient for commercial use. Through compilation, the main use of angelicin described in patents for pharmaceutical drugs are for thalassemia and sickle cell treatment, vaccines for herpes virus and preparation material for hyperlipemia medicine (Bianchi et al., 2003; Bianchi et al., 2008; Song et al., 2010; Spector et al., 2012; Li et al., 2017b). Through the brief description of the patents, angelicin was able to decrease the content of total cholesterol, low-density lipoprotein cholesterol, and triglycerides in rats. It can also increase high-density lipoprotein cholesterol (Li et al., 2017b). Angelicin has also been patented as an active ingredient for a herbicide component (Kim et al., 2009). Through these patents, it can be seen how under-researched angelicin is even though it demonstrates great potential in *in vitro* and *in vivo* studies.

## Future New Research Horizons

For future prospects, studies on improving angelicin drug delivery, such as the use of nanoparticles should be done to increase its delivery efficacy while reducing unwanted side effects. For example, angelicin can be encapsulated in nanoparticles to improve its anti-cancer properties. In a study done by Boondireke et al. (2019), hydrophobic monomyristin encapsulated in dextran-covered polylactide nanoparticle, which had a superficial layer of conjugated protein transferrin, had showed increased cytotoxicity against HeLa cells in comparison to non-encapsulated monomyristin. Another method was utilizing photosensitive nanoparticles to change the microenvironment of tumors, making it more susceptible to anti-cancer treatments. To combat tumor hypoxia, Lan et al. (2018) developed novel nanoscale metal-organic based nanophotosensitizers, Fe-TBP. Fe-TBP was made from 5,10,15,20-tetra(p-benzoato)porphyrin (TBP) and Fe_3_O clusters, which catalyzes intracellular H_2_O_2_ to O_2_ *via* Fenton-like reaction when irradiated. The O_2_ was then converted by photoexcited porphyrins to cytotoxic singlet oxygen (^1^O_2_). The end results were significant improvement of anti-programmed death-ligand 1 (α-PD-L1) treatment efficacy *via* the increasing tumor infiltration by cytotoxic T cells, prompting abscopal effects in mouse colorectal cancer. In a recent study, psoralen that had been encapsulated in polymer -lipid hybrid nanocarrier had not only displayed improved water solubility but also displayed an increased inhibitory effect against MCF-7 tumors *in vivo* as compared to non-encapsulated psoralen. This is due to the passive accumulation of encapsulated psoralen in the tumor and extended circulation time, which brings about a lengthened exposure of the psoralen toward the tumor (Du et al., 2019). Gut microbes also metabolize the consumed psoralen and angelicin, therefore encapsulation might improve the transfer of angelicin to the targeted site (Liu et al., 2019). Similar usage of nanoformulations containing arsenic sulphide had also been used on chronic myeloid leukemia cells to induce erythroid differentiation (Wang et al., 2019a), while Pabari et al. (2019) had utilized biodegradable polyesterurethane and PEGylated polymers to form nanocarriers of infliximab in the treatment against inflammatory cytokines in monocytes for 24 h. As can be seen, nanoparticles or nanoformulations can mediate a more targeted, effective and controlled release of drugs. It can also be used to improve the microenvironment in deep parts of tumors to increase the efficacy of drugs. Hence, its incorporation with anti-cancer, anti-inflammatory and erythroid differentiation angelicin studies should be considered.

In addition, research on the effect of angelicin on osteoclasts and other disease models, such as bone fractures and osteoarthritis, should be done as well. Psoralen had been reported to speed up the healing of fractures on the tibia of rats by activating osteoclasts and osteoblasts through ERK signaling (Zhang et al., 2019). As psoralen and angelicin are isomers of each other, it would prove interesting to see the effect of angelicin on bone fractures. Lately, there was a publication reporting that neural EGFL like 1 (NELL-1)-haploinsufficient mice displayed increased inflammatory markers and symptoms of aggravated and accelerated osteoarthritis (Li et al., 2020). Li et al. (2020) had also reported that NELL-1 mediates a decrease of inflammatory marker IL1β by increasing the Runx1 gene in chondrocytes. Therefore, there is a possibility that angelicin might be able to reverse the effect of osteoarthritis as previous studies had shown that angelicin increases Runx2 transcription and also has anti-inflammatory properties. To confirm this hypothesis, further studies of angelicin on osteoarthritis model needs to be done. On another note, periodontitis is not only caused by persistent microbial infections but also viral infections by the *Herpesviridae* family of viruses (Chen et al., 2020). Saliva samples of patients with apical periodontitis had detected the presence of EBV and HCMV (Jakovljevic et al., 2018). With angelicin having anti-viral properties, the effect of angelicin on the virus population found in periodontitis can be explored. Different virus strains too should be tested against angelicin to see if angelicin is able to inhibit a wide range of viruses.

As previously reported, angelicin forms monoadducts in the DNA under UV irradiation. Studies have shown modulation of transcription factors such as NF-κB and Runx after the application of angelicin. It is possible that these changes in expression could be due to the interaction of angelicin with DNA or RNA molecules. However, this has yet to be elucidated and more in-depth studies are needed. Interestingly, a study on 4,6,4-trimethylangelicin (4,6,4-TMA), a derivative of angelicin, showed inhibitory effects on NF-κB/DNA interaction. Using electrophoretic mobility shift assay, the angelicin analogues successfully suppressed the interaction of NF-κB p50 and double-stranded oligonucleotides, which was designed to mimic the NF-κB consensus sequence (Lampronti et al., 2017). To verify if angelicin can also directly inhibit the binding of NF-κB to DNA, experimental research, molecular docking, and molecular dynamics simulation studies can be performed to understand their interaction. Similarly, the interaction of angelicin with other regulatory genes, transcription factors and receptors could be explored in future research.

## Conclusion

In summary, angelicin shows great promise in various aspects especially in its anti-cancer, anti-inflammatory, and pro-osteogenic properties. Besides that, further studies involving the drug delivery of angelicin may improve the effect of angelicin toward the targeted sites in cancer, inflammation and erythroid differentiation models. A deeper understanding of the effect of angelicin in the mechanism of bone modeling and other bone-related models are also needed. Other than that, knowledge on the anti-viral implications of angelicin in periodontitis and the interaction of angelicin with regulatory genes can be expanded.

## Author Contributions

The writing was performed by CM, LT, PP, LHL, and BG. While WY, WL, LHL, ST, KC, LEL and BG provided vital guidance and insight to the work. The project was conceptualized by BG.

## Funding

This work was inspired by Monash Pharmacy Degree Course, Unit PAC3512 which entitled “Current aspects of pharmaceutical research” and financially supported by Taylor's University Emerging Grant (TRGS/ERFS/2/2018/SBS/016), University of Malaya Research Grant (FRGS grant to KGC grant no: FP022-2018A), External Industry Grant from Biotek Abadi Sdn Bhd (vote no. GBA-81811A), Monash Global Asia in the 21st Century (GA21) research grants (GA-HW-19-L01 & GA-HW-19-S02), and Fundamental Research Grant Scheme (FRGS/1/2019/WAB09/MUSM/02/1 & FRGS/1/2019/SKK08/MUSM/02/7).

## Conflict of Interest

The authors declare that the research was conducted in the absence of any commercial or financial relationships that could be construed as a potential conflict of interest.
